# Metabolomics insights into the modulatory effects of long-term compound polysaccharide intake in high-fat diet-induced obese rats

**DOI:** 10.1186/s12986-018-0246-2

**Published:** 2018-01-23

**Authors:** Mingyi Chen, Biyu Lu, Yuan Li, Yuanyuan Wang, Haihui Zheng, Danmin Zhong, Ziqiong Liao, Mengxia Wang, Fangli Ma, Qiongfeng Liao, Zhiyong Xie

**Affiliations:** 1 School of Pharmaceutical Sciences, Sun Yat-sen University, Guangzhou, 510006 People’s Republic of China; 2 School of Pharmaceutical Sciences, Guangzhou University of Chinese Medicine, Guangzhou, 510407 People’s Republic of China; 3 Infinitus (China) Company Ltd, Guangzhou, 510623 China

**Keywords:** Obesity, Metabolomics, NMR, Lentinan, *Flos Lonicera* polysaccharides(FLP)

## Abstract

**Background:**

Polysaccharides can alleviate obesity in mammals; however, studies on mechanism of this alleviation are limited. A few studies have indicated that polysaccharides improve obesity by regulating the metabolism of the body. Therefore, a metabolomics approach, consisting of high resolution nuclear magnetic resonance (NMR) spectroscopy and a multivariate statistical technique, was applied to explore the mechanism of the protective effects of lentinan and *Flos Lonicera* polysaccharides (LF) on high-fat diet (HFD) induced obesity.

**Methods:**

In this study, rats were randomly divided into three groups: control diet (CD), HFD, and HFD supplemented with a mixture of lentinan and *Flos Lonicera* polysaccharide. Histopathological and clinical biochemical assessments were also conducted. A combination of a NMR metabolomics study and a multivariable statistical analysis method to distinguish urinary and fecal metabolites was applied.

**Results:**

Significant obesity symptoms appeared in HFD rats (for example, significant weight gain, epididymal adipose accumulation and lipid deposition in hepatocytes), which was attenuated in the LF group. Additionally, the HFD induced a reduction of choline, citrate, pyruvate and glycerol and increased the levels of trimethylamine oxide (TMAO) and taurine. Of note, these metabolic disorders were reversed by LF intervention mainly through pathways of energy metabolism, choline metabolism and gut microbiota metabolism.

**Conclusions:**

LF supplementation had a re-balancing effect on the disturbed metabolic pathways in the obese body. The results of this study validate the therapeutic effect of the compound polysaccharide--LF in obesity and described the biochemical and metabolic mechanisms involved.

**Electronic supplementary material:**

The online version of this article (10.1186/s12986-018-0246-2) contains supplementary material, which is available to authorized users.

## Background

Obesity, which was officially declared a disease by the World Health Organization (WHO) in 1997 [1], is associated with a cluster of metabolic disorders such as hyperlipidemia, hypertension, diabetes, coronary-heart diseases, and certain cancers [2]. Obesity not only affects the well-being of a person, but also places an unwanted economic burden on society [3]. Therefore, it is necessary urgent to find an effective way to prevent and treat obesity. Growing evidence has indicated that obesity is closely linked with metabolic disturbance and chronic, low-grade inflammation, and we can improve obesity by reversing metabolic disorders [4]. Accordingly, probiotics and prebiotics are considered to be important assets in the management of obesity and its associated metabolic syndrome because they can modulate the metabolism of the host in a positive way [5, 6]. Polysaccharides, a type of prebiotics, had shown functions to reduce body weight and fat accumulation [3], lower serum glucose [7] and have protective effects on the liver [8]. For example, Chang et al. [7] discovered that *Ganoderma lucidum* reduced body weight, fat accumulation, inflammation, endotoxemia and insulin resistance in obese mice and prevented obesity-related metabolic disorders in obese individuals. Additionally, it was also reported that MDG-1, a β-D-fructan polysaccharide extracted from *Ophiopogon japonicus*, could prevent the development of obesity and ameliorate dyslipidemia in high-fat diet (HFD)-induced obese mice [9, 10]. Interestingly, a compound polysaccharide, which is a mixture of lentinan and *Flos Lonicera* polysaccharides (LF), improved obesity and its related complications in our research studies (unpublished data). Lentinan, a well-known medicinal fungal polysaccharide, is a glucan produced by the edible mushroom *Lentinus* (or *Lentinula*) *edodes*, and has been widely used as a component of traditional diets and medicine in Asia for hundreds of years [11]. Previous studies have shown that lentinan has hypolipidemic and hypoglycemic effects in cell culture experiments [12, 13]. *Flos Lonicera*, another widely used medicinal and edible herb, also has hepatoprotective, antihypertensive, anti-inflammatory, antioxidative and antiviral activities [8, 14, 15], and *Flos Lonicera* polysaccharides (FLP) was reported to have a wide range of pharmacological effects, such as antioxidant and antiallergy effects [16, 17]. Despite the beneficial health effects of lentinan and FLP described above, few scientific studies have been conducted to elucidate the possible mechanisms by which this compound polysaccharide may exert its protective effects against obesity and its associated metabolic disorders. Thus, we aimed to evaluate the therapeutic effect of the compound polysaccharide lentinan and FLP on rat obesity.

Recently, developed metabolomics tools have allowed researchers to better probe the progression of diseases, elucidate their pathogenesis, and assess the effects of health products on certain pathologies [18]. Numerous studies have shown that ^1^H nuclear magnetic resonance (NMR)-based metabolomics is a promising technique for metabolomics study and can provide a rapid and non-invasive analysis [19, 20]. Additionally, a practicable ^1^H NMR-based metabolomics method has also been established and applied successfully to observe and analyze metabolite changes in organisms in previous researches by our group [21, 22]. In the present study, we aimed to explore the effects of lentinan and FLP treatment on obesity to elaborate on the mechanism via metabolomics.

In the current study, a 12-week investigation was performed to identify the time-dependent characteristics of metabolic responses including a 4-week HFD-feeding and 8-week mixed polysaccharides intervention in rats. We applied the NMR-based metabolomics approach to assess the preventive effects of lentinan and FLP on HFD-induced obesity in rats, which may elucidate the possible mechanisms by which this compound polysaccharide exerts its protective effects against obesity and associated complications.

## Methods

### Chemicals and animal diets

Potassium phosphate dibasic (K_2_HPO_4_·3H_2_O), sodium phosphate monobasic (NaH_2_PO_4_·2H_2_O) and sodium azide (NaN_3_) (all of analytical grade) were purchased from Tianjin Da Mao Chemicals Reagent Factory (Tianjin, China). D_2_O (with 0.05% sodium 3-trimethylsilyl-(2,2,3,3-^2^H_4_)-1-propionate, TSP) was obtained from Sigma-Aldrich Trading Co., Ltd. (Shanghai, China). A mixture of lentinan and *Flos Lonicera* polysaccharide (1:1, *wt*/*wt*) was generously provided by Infinitus (China) Company, Ltd. (Guangzhou, China). The control diet (CD, 10% kcal from fat) and HFD (40% kcal from fat) were adapted from those of Research Diet, Inc. (New Brunswick, NJ). The HFD supplemented with a mixture of lentinan and *Flos Lonicera* polysaccharide (LF, 0.675%, *wt*/*wt*) was prepared by Beijing HFK Bioscience Co., Ltd. (Beijing, China). More specifically, for example, 1000 g HFD and 6.75 g LF were combined to obtain 1006.75 g HFD + LF, which was used as the diet of rats in LF group. The composition of the diets is shown in Additional file 1: Table S1.

### Ethics statement

All animal experimental procedures were performed at the Sun Yat-sen University Animal Experiment Center (Guangzhou, China). The protocol was reviewed and approved by the Institutional Animal Care and Use Committee (IACUC) of Sun Yat-sen University, and conformed to the National Institute of Health guidelines on the ethical use of animals. All efforts were made to ameliorate animals suffering.

### Animal treatment and sample collection

Male Sprague-Dawley (SD) rats (weighting 160–180 g and a median of 173 g, 6 weeks old) were supplied by the medical laboratory animal center of Guangdong province (Guangzhou, China) and kept in a specific pathogen free (SPF) experimental room (12 h light/dark cycle, 20 °C, 50%–70% humidity) with free access to food and water. After acclimatization for 7 days, a total of 40 rats were randomly divided into 2 groups, labelled the control diet group (CD group, *n* = 10) and high fat diet group (*n* = 30). After 4 weeks, the HFD induced obesity resistant rats, those who gained weight in the lower quartiles were not further studied [23, 24], while the remaining rats were randomized again into 2 groups: one group was continuously maintained on the HF diet (HFD group, *n* = 10) and the other group was fed the LF diet (LF group, *n* = 10) for 8 weeks. Rats in the CD group were fed the CD diet throughout the trial.

The animal experiment lasted for 12 weeks, during which the food intake and body weights were recorded daily and weekly, respectively. Fresh urine and fecal samples were collected (from 8:00 am to 16:00 pm) at pretreatment day − 1 and at weeks 4, 8, and 12 using metabolic cages with ice-packed Eppendorf tubes and immediately stored at − 80 °C until analysis. At the end of the trial, all of the animals were anaesthetized after an overnight fast. Serum samples were collected from the orbital plexus while liver and epididymal adipose tissues were harvested for further analysis.

### Histopathological and clinical biochemical assessments

For histopathological assessments, frozen liver sections (6 mm thick) were stained with Oil Red O (Sigma, USA) for 20 min. Examinations were performed out blindly by an experienced pathologist of Sun Yat-sen University Animal Experiment Center under microscopy.

Clinical chemistry analyses including total serum cholesterol (TC), triglycerides (TG), high-density lipoprotein cholesterol (HDL-C), low-density lipoprotein cholesterol (LDL-C), alanine aminotransferase (ALT), aspartate aminotransferase (AST) and serum glucose (Glu) were conducted with the standard routine procedures on a Beckman CX5 automatic biochemical analyzer (Beckman Coulter, Inc., USA) in the clinical laboratory of Sun Yat-sen University Animal Experiment Center.

### Sample preparation for NMR spectroscopy

Urine samples were removed from − 80 °C storage and thawed at room temperature. An optimized method was adopted for preparing the samples. Urine samples (550 μL) were mixed with 55 μL of a phosphate buffer solution (K_2_HPO_4_/NaH_2_PO_4_, 1.5 M, pH 7.4, 100% D_2_O) containing 0.1% NaN_3_ and 0.05% TSP [25]. After centrifugation at 16000×*g* for 10 min at 4 °C, 550 μL of the supernatant was pipetted into 5 mm NMR capillary tubes for NMR analysis. Fecal extraction referred to the optimized method described by Wu et al. [26]. The details are as follows: Fecal extraction was performed on ice by adding 800 μL of phosphate buffer (0.1 M, pH 7.4) to 100 mg of thawed stool sample. After vortex mixing for approximately 1 min, the mixed slurry is underwent freeze-thaw treatments (3 times), followed by 10 ultrasonication cycles to optimize the extractions. Ultrasonication was conducted in an ice bath in the form of ultrasonication (20 s)-vortex (10 s)-waiting (30 s). After a 10 min centrifugation (16,000×*g* at 4 °C), 550 μL of the supernatant was withdrawn, followed by ^1^H NMR measurements.

The proton spectra of the samples were acquired at 298 K on a Bruker AVIII 600 MHz spectrometer operating at 600.13 MHz. The acquisition parameters were similar to the previous report [27]. A one-dimensional pulse sequence was collected using the NOESY pulse sequence [recycle delay(RD)-G_1_–90°-t_1_–90°-t_m_-G_2_–90°-acquisition] with irradiation during a 2 s relaxation delay and during the 80 ms mixing time to suppress the water signal. Generally, a total of 64 transients for urine samples were collected into 32 k data points over a spectral width of 20 ppm with a 90° pulse length adjusted to approximately 10 μs for all of the samples.

For signal assignment purposes, two-dimensional (2D) NMR experiments, including ^1^H-^1^H Total Correlation Spectroscopy (TOCSY), ^1^H-^1^HCorrelation Spectroscopy (COSY), ^1^H-^13^C Heteronuclear Single Quantum Correlation (HSQC) and Heteronuclear Multiple Bond Correlation spectroscopy (HMBC), were acquired for selected samples as previously reported [21, 22, 28].

### NMR data processing

The ^1^H NMR spectra of urine and fecal extracts were corrected for phase and baseline distortion using TOPSPIN (V3.2, Bruker Biospin, Germany) and referenced to the TSP resonance at *δ* 0.00. The urinary and fecal spectral region *δ* 0.50–9.50 was integrated into regions with an equal width of 0.004 ppm using an AMIX software package (V3.2, Bruker Biospin, Germany). For urinary spectra, the water signal (*δ* 4.75–4.90) and urea signal (*δ* 5.60–6.00) were removed prior to analysis. For fecal samples, the water signal (*δ* 4.75–4.90) was also discarded. The spectral data were normalized to the total spectral area prior to data analysis. The metabolite resonances were assigned on the basis of comparison with the literature and existing databases, such as the Human Metabolome Database (HMDB: http://www.hmdb.ca/), and metabonomics toolbox (Chenomx NMRSuit 7.6, Chenomx, Canada).

### Statistical analysis

Multivariate data analysis was conducted with SIMCA-P+ (V12.0, Umetrics, Sweden) and statistical product and service solutions (SPSS) software (V19.0, Chicago, USA). Body weight, body composition and clinical biochemical parameter data are presented as the means ± standard error and were processed using one way ANOVA followed by the Tukey post hoc analysis test with a critical level of *p* < 0.05. Principal Component Analysis (PCA) was performed on Pareto variance (Par)-scaled data to monitor possible outliers in each matrix. Orthogonal projection to latent structures discriminant analysis (OPLS-DA) was then performed to obtain the metabolites that made significant contributions to the intergroup differentiation with Ctr-scaled NMR data as the X-matrix and class information and as the Y-matrix. All of the OPLS-DA models were validated by 7-fold cross-validation and ANOVA of the cross validated residuals (CV-ANOVA) at a level of *p* < 0.05 [29, 30]. Using the web-based analysis tool, Metaboanalyst (www.metaboanalyst.ca), receiver operating characteristic (ROC) curves were drawn to assess the robustness of the models. ROC analyses were based on partial least squares discrimination analysis (PLS-DA) models as classification methods with 3 latent variables. The model sensitivity and specificity were calculated from the ROC confusion matrix (generated on the basis of the average of the predicted class probabilities of each sample across 100 cross-validations). ROC curves were generated by Monte-Carlo cross validation (MCCV) using balanced sub-sampling. In each MCCV, two-thirds (2/3) of the samples were used to evaluate the feature importance. The top 100 important features were then used to build classification models that were validated on the remaining 1/3 sample. The procedure was repeated multiple times to calculate the performance and confidence intervals of each model. A new feature of OPLS-DA is S-plot [31], which was used to established a model visualization. OPLS-DA and S-plots are typically used to identify significant biomarkers to distinguish between two groups. In addition to the multivariate statistical method, significant *p*-values associated with altered metabolites were corrected for multiple hypotheses testing using the Benjamini-Hochberg false discovery rate (FDR) method. Metabolomics Pathway Analysis (MetPA), a web-based pathway analysis and visualization tool (www.metaboanalyst.ca), was introduced to help identify the most relevant metabolic pathways based on the protocol [32, 33].

## Results

### Effects of LF on high-fat diet induced obese rats

**Fig. 1:**
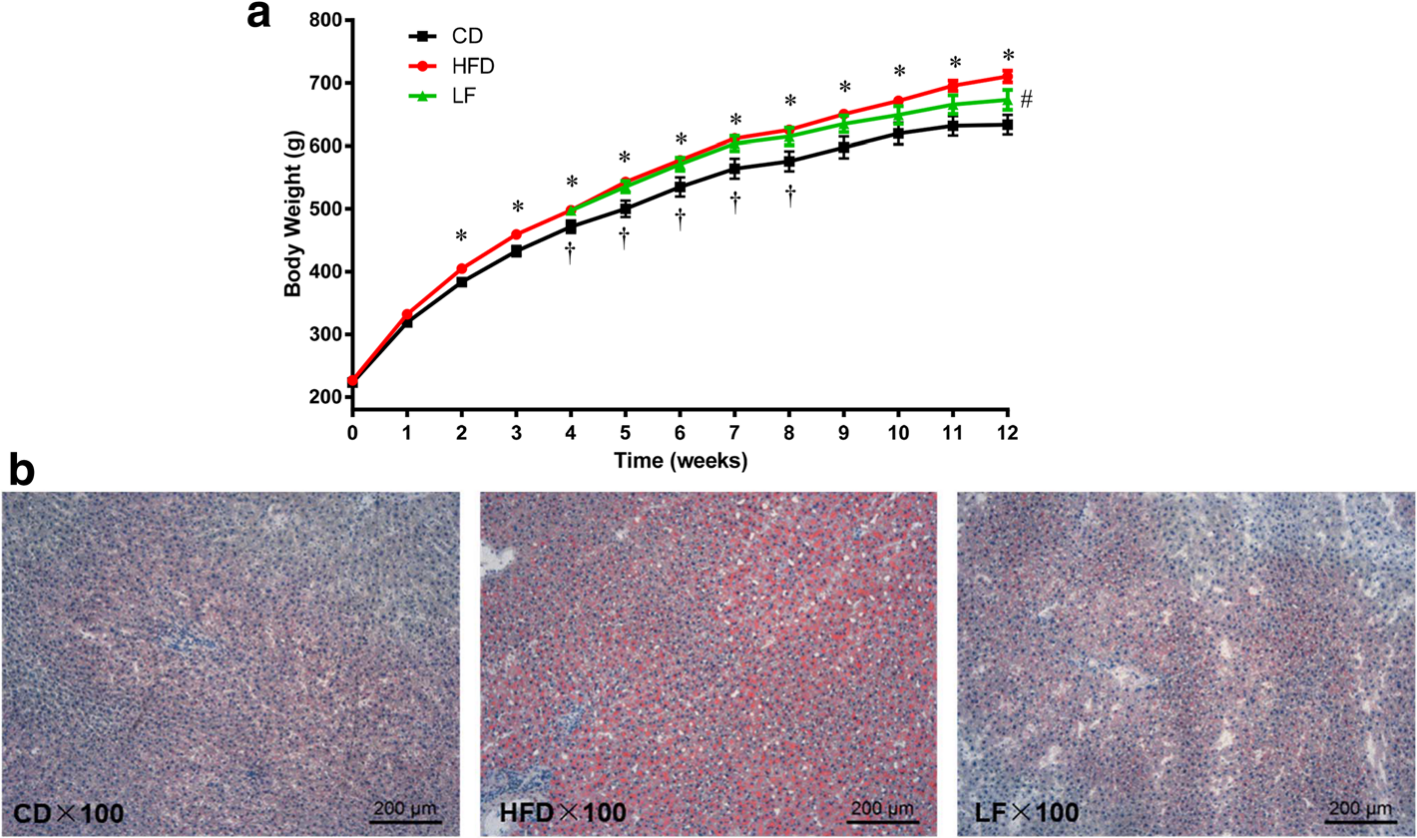
Effects of LF on body weight gain and the morphology of the liver in HFD-fed rats. **a** Body weight gain; **b** Micrographs of rat liver specimens from CD, HFD, and LF treatment groups. Values are expressed as means ± standard error. Differences were assessed by ANOVA and denoted as follow: values are statistically significant at *p* < 0.05; *, significantly different from the CD group; #, significantly different from the HFD group; †, significantly different from the LF group (*n* = 10)

Throughout the entire 12 weeks period, HFD feeding led to significant increases in body weight, epididymal adipose accumulation, and lipid deposition in hepatocytes compared with control diet feeding (Additional file 1: Figure S1, Fig. 1). HFD supplied with LF showed a trend in decreasing body weight gain and the accumulation of fat. Lipid deposition in hepatocytes was notably decreased in the LF-treatment group compared with the HFD group without LF intervention (Fig. 1). However, there were no difference in mean energy intake between LF and HFD groups (Additional file 1: Figure S1-D), suggesting that the effects of LF in the results mentioned above were not due to reduced food consumption or energy extraction.

**Fig. 2:**
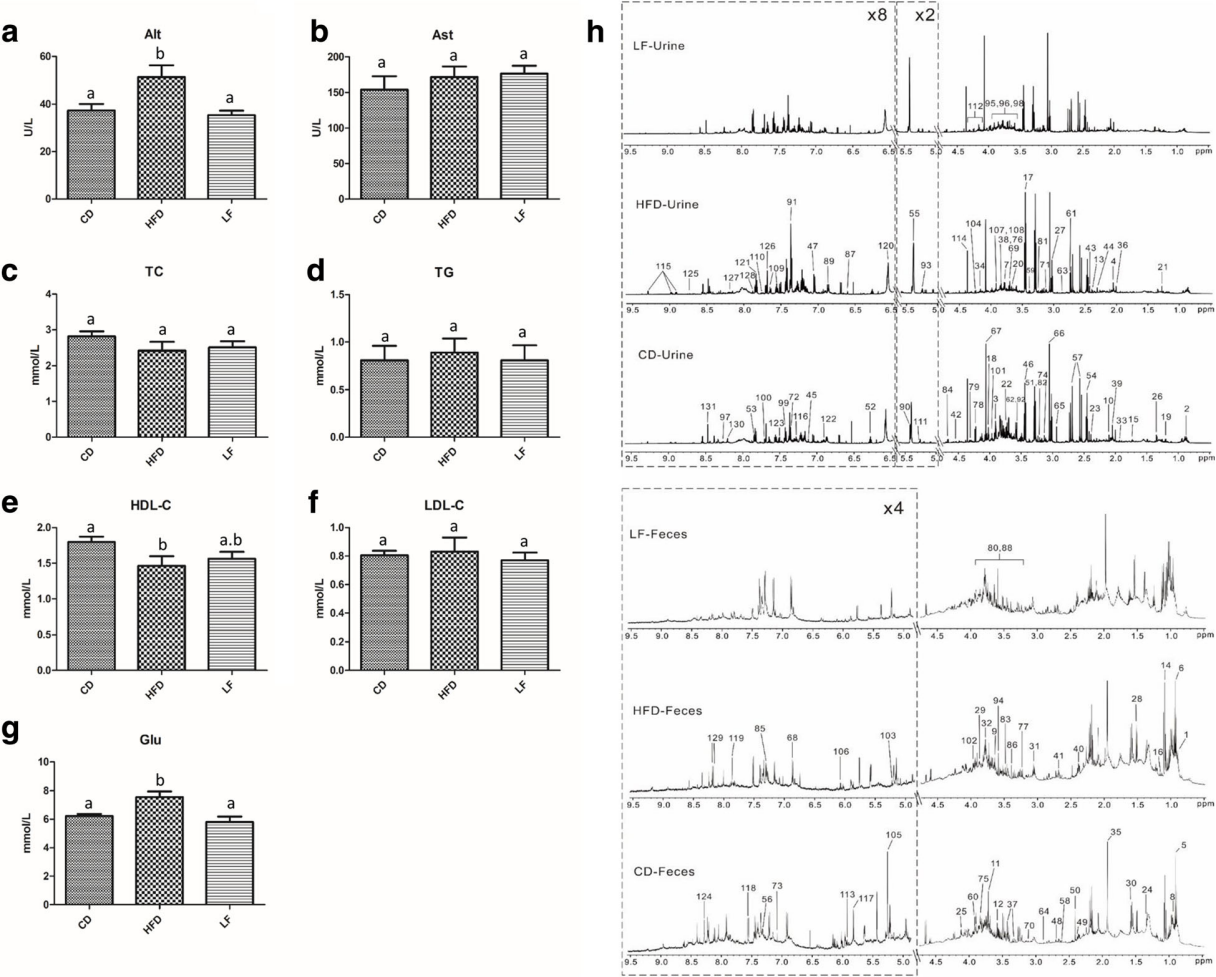
Influences of LF on the liver, blood lipids and serum glucose in HFD-fed rats, and typical 600 MHz ^1^H NMR spectra of urine and feces. **a** Serum Alt; **b** serum Ast; **c** serum TC; **d** serum TG; **e** serum HDL-C; **f** serum LDL-C; **g** serum Glu; **h** typical 600 MHz ^1^H NMR spectra of urine and feces from CD, HFD and LF treated rats at 12 week. Abbreviations: Alt, alanine aminotransferase; Ast, aspartate aminotransferase; TC, total cholesterol; TG, total triglycerides; HDL-C, high-density lipoprotein cholesterol; LDL-C, low-density lipoprotein cholesterol; Glu, glucose. Values are expressed as means ± standard error. Graph bars with different letters on top represent statistically significant results (*p* < 0.05) based on one-way ANOVA analysis, whereas bars with the same letter correspond to results that show no statistically significant differences. In the case where two letters are present on top of the bars in (**e**), each letter should be compared separately with the letters of other bars to determine whether the results show statistically significant differences

For clinical biochemical assessments, HFD caused a marked elevation of Glu and Alt accompanied by a significant HDL-C decrease (Fig. 2a–g). However, with LF treatment for 8 weeks, the levels of Glu and Alt were notably decreased while the HDL-C level was increased. These results indicated that LF had hypoglycemic, hypolipidemic and hepatoprotective effects. Throughout the entire experiment, serum TG, TC, LDL-C and Ast showed no significant differences among the three groups (Fig. 2b-d, f).

### NMR spectra of rat urine and feces extracts

Representative ^1^H NMR spectra of urine and fecal samples from the CD, HFD and LF group showed rich metabolite information related to 12-week differential diet interventions (Fig. 2h). The metabolites involved in the spectra were assigned based on previously published work [21, 22, 28] and further confirmed by 2D NMR spectra. A total of 103 metabolites in urine samples and 53 components in fecal solutions were detected (Additional file 1: Table S2). The NMR spectrum showed that obvious level changes in urinary phenylacetate, phenylalanine, creatine and creatinine as well as arabinose, pyrimidines and organic acids in fecal extracts were caused by different diets (Fig. 2h). To obtain more details on the metabolomics changes, multivariate data analysis was conducted on the NMR data for urine and fecal extracts.

### Metabolic patterns analysis of urine and feces in obese rats treated with LF

**Fig. 3:**
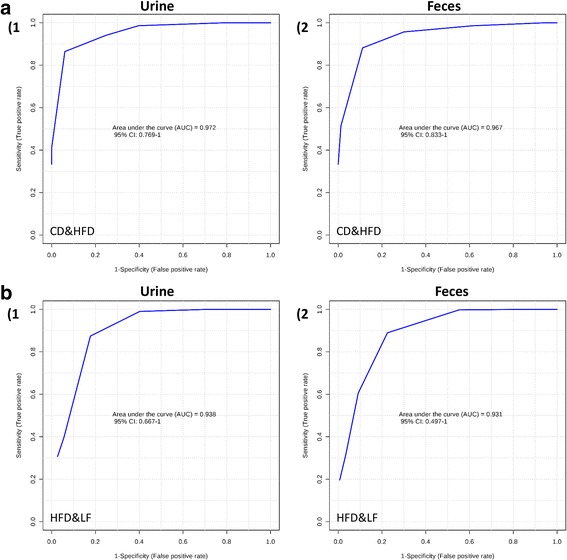
Receiver operating characteristic (ROC) curves of ^1^H NMR metabolomics of urine and fecal samples. Multivariate ROC curves were drawn using ^1^H NMR metabolomic spectral data from the corresponding groups. (**a**-1) urine samples from HFD & CD; (**a**-2) fecal samples from HFD & CD; (**b**-1) urine samples from LF & HFD; (**b**-2) fecal samples from LF & HFD

PCA was initially applied to the NMR data obtained from the urine and fecal samples. A PCA score plot showed clear differences among the CD, HFD and LF groups in their metabolite profiles of urine extracts (Additional file 1: Figure S2-A). Nevertheless, the discrimination between the HFD and LF groups in the fecal extracts was not as obvious as in their urinary counterparts (Additional file 1: Figure S2-B). However, pairwise comparisons, whether between the CD and HFD or between the HFD and LF groups, presented clear classifications both in urine and fecal extracts (Additional file 1: Figure S2). OPLS-DA was subsequently employed to identify the metabolites that were altered after different diet interventions. The R^2^ and Q^2^ values of all of the mathematical models are summarized in Additional file 1: Tables S3 and S4. CV-ANOVA demonstrated that all of the models were valid and suitable for data-mining (Additional file 1: Tables S3 and S4). Model validity was assessed by ROC analysis (based on PLS-DA model), as described above. The sensitivity and specificity of the model are shown in Additional file 1: Table S5. The area under the ROC curve (AUC) between CD and HFD in urine and feces were 0.972 and 0.967, respectively, suggesting a high predictive accuracy (Fig. 3). Additionally, the areas under the ROC curves (AUCs) between HFD and LF in urine and feces were 0.938 and 0.931, respectively.

### Metabolic changes in urine and feces in obese rats treated with LF

**Fig. 4:**
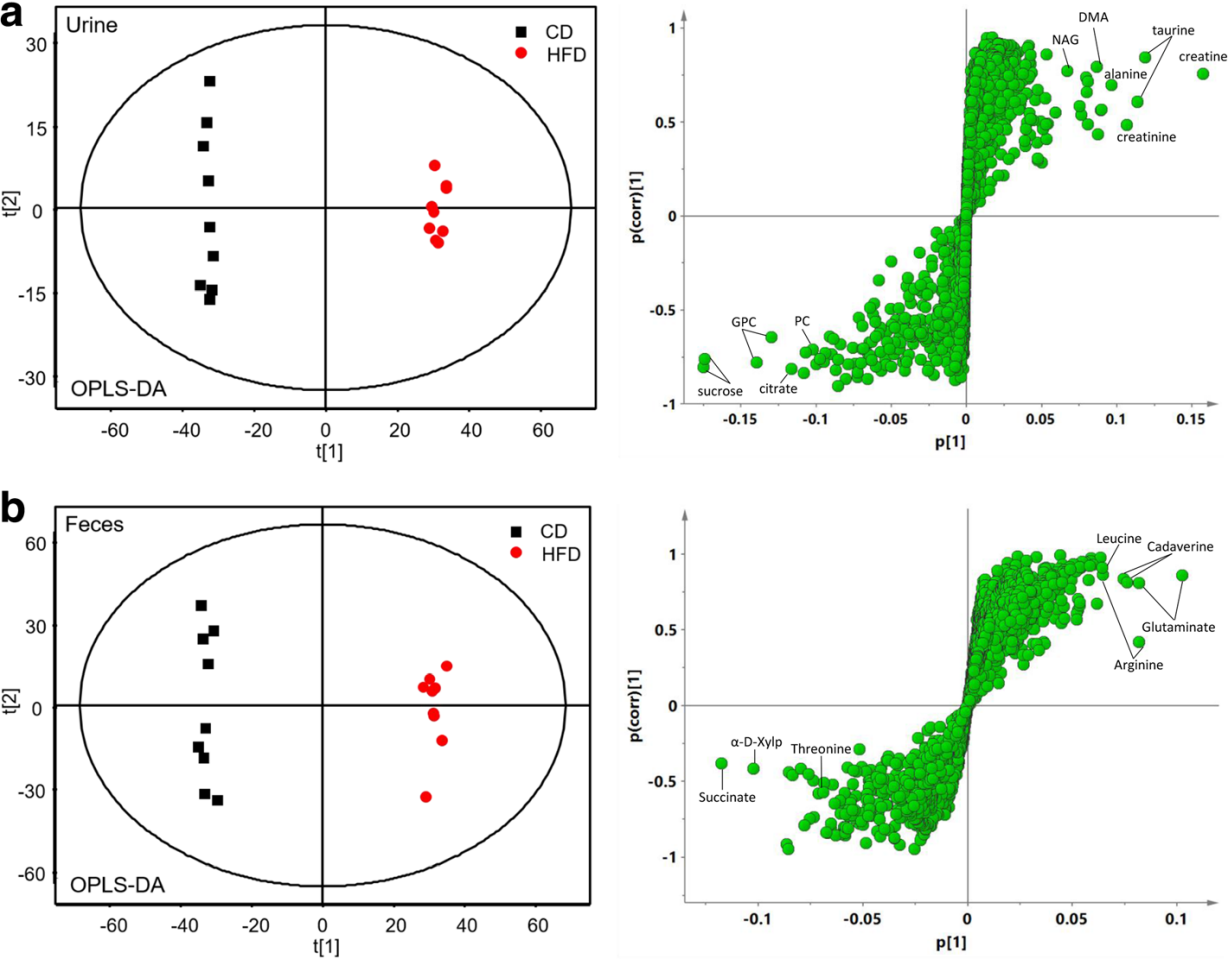
**a** Urine OPLS-DA score plots and S-plots of the CD and HFD group based on ^1^H NMR technology (HFD vs. CD). **b** Feces OPLS-DA score plots and S-plots of the CD and HFD group based on ^1^H NMR technology (LF vs. HFD). Some makers were labeled in the S-plots. Abbreviations: NAG, N-acetyl-beta-D-glucosaminidase; DMA, Dimethylamine; GPC, Glycerophosphocholine; PC, phosphorylcholine; α-D-Xylp, α-D-xylopyranosyl

The clear classifications between the CD and HFD groups are shown in the OPLS-DA score plots (Fig. 4), while the S-plots illustrated significantly altered metabolites induced by the HFD in urine and feces samples. The urine results revealed that HFD feeding for 12 weeks induced a significant elevation of urinary acetoacetate, creatine, creatinine, allantoin, phenylacetate, hippurate, phenylalanine, succinimide, N-acetyl-beta-D-glucosaminidase (NAG), N-acetylglutamate, uracil, valine, levulinate, alanine, 2-methylglutarate, 4-cresol, leucine, trimethylamine, 2-hydroxybutyrate, N-phenylacetylglycine and glycogen accompanied with decreased levels of phosphorylcholine, ornithine, N-nitrosodimethylamine, glycerol, citrate, betaine, sucrose, glycine, glycerophosphocholine and propylene glycol in urine (Fig. 4, Table 1). Moreover, the fecal levels of imidazole, urocanate, 3-phenylpropionate, glutamate, phenylacetate, tyrosine, 2-oxoglutarate, cadaverine, valine, leucine, 5-aminovalerate, uracil, arginine, creatine, malonate and α-ketoisocaproate were significantly elevated in HFD-fed rats, while threonine, lactate and α-arabinose were reduced in this group (Fig. 4, Table 2). Besides, the content changes of these significant altered metabolites between rats in CD, HFD and LF group were shown in Additional file 1: Tables S6 (in urine) and S7 (in feces).

**Table 1:** Statistical analysis results of the main metabolite change in urine

Metabolites	Group HFD vs CD			Group LF vs HFD		
	*p* value†	Adjust *p* value‡	Trend	*p* value†	Adjust *p* value‡	Trend
Acetoacetate	< 0.0001	< 0.0001	↑**	0.0085	0.00173	↓**
Creatine	< 0.0001	< 0.0001	↑**			
Creatinine	< 0.0001	< 0.0001	↑**			
Allantoin	< 0.0001	0.0002	↑**			
Phenylacetate	0.0022	0.0030	↑**			
Hippurate	< 0.0001	0.0002	↑**	< 0.0001	< 0.0001	↑**
Phenylalanine	0.0023	0.0029	↑**			
Succinimide	< 0.0001	0.0002	↑**			
N-Acetylglutamate	0.0001	0.0003	↑**			
Uracil	0.0003	0.0006	↑**			
Valine	< 0.0001	< 0.0001	↑**			
Levulinate	0.0008	0.0012	↑**			
Alanine	0.0020	0.0030	↑**			
2-Methylglutarate	0.0002	0.0004	↑**			
*p*-cresol	0.0005	0.0008	↑**			
Leucine	0.0021	0.0029	↑**			
Trimethylamine	0.0027	0.0032	↑**			
2-Hydroxybutyrate	0.0132	0.0146	↑*			
Glycogen	0.0025	0.0031	↑**			
Phenylacetylglycine	0.0373	0.0383	↑*			
Dimethylamine	0.0024	0.0030	↑**			
Carnitine	< 0.0001	< 0.0001	↑**			
Taurine	0.0001	0.0003	↑**			
Tryptophan	< 0.0001	< 0.0001	↑**			
2PY	< 0.0001	< 0.0001	↑**			
Guanine	0.0004	0.0007	↑**			
Nicotinamide	0.0068	0.0083	↑**			
Histidine	0.0160	0.0172	↑*	0.0283	0.0367	↓*
Phosphorylcholine	0.0318	0.0344	↓*			
Ornithine	0.0097	0.0111	↓**			
N-Nitrosodimethylamine	0.0324	0.0341	↓*	0.0371	0.0402	↑*
Glycerol	0.0021	0.0029	↓**	0.0003	0.0018	↑**
Citrate	0.0398	0.0398	↓*			
Betaine	0.0004	0.0007	↓**			
Sucrose	0.0005	0.0008	↓**			
Glycine	< 0.0001	< 0.0001	↓**			
Glycerophosphocholine	< 0.0001	< 0.0001	↓**			
Propylene glycol	< 0.0001	< 0.0001	↓**			
Galactonate	< 0.0001	< 0.0001	↓**			
Pseudouridine	< 0.0001	0.0002	↓**			
Fumarate	0.0002	0.0004	↓**			
4PY				0.0005	0.0015	↑**
Ethanol				0.0011	0.0029	↑**
N-Methylhydantoin				0.0175	0.0285	↑*
Sarcosine				0.0025	0.0055	↑**
Xanthine				0.0291	0.0344	↑*
Acetamide				0.0243	0.0351	↓*
Pyruvate				0.0133	0.0246	↓*
Formate				0.0408	0.0408	↓*
Trimethylamine N-oxide				0.0003	0.0013	↓**

*Abbreviations*: *2PY* N′-methyl-2-pyridone-5-carboxamide, *4PY* N′-methyl-4-pyridone-3-carboxamide

†From Student t test. *p < 0.05; **p < 0.01. ‡Adjusted by false discovery rate method across the metabolites within each comparison. HFD vs CD: compare HFD to CD; LF vs HFD: compare LF to HFD

**Table 2:** Statistical analysis results of the main metabolite change in feces

Metabolites	Group HFD vs CD			Group LF vs HFD		
	*p* value†	Adjust *p* value‡	Trend	*p* value†	Adjust *p* value‡	Trend
Imidazole	0.0002	0.0006	↑**			
Urocanate	< 0.0001	< 0.0001	↑**	0.0197	0.0395	↑*
3-Phenylpropionate	< 0.0001	< 0.0001	↑**	0.0245	0.0429	↑*
Glutamate	< 0.0001	0.0001	↑**			
Phenylacetate	< 0.0001	< 0.0001	↑**	0.0268	0.0418	↑*
Tyrosine	< 0.0001	< 0.0001	↑**	0.0017	0.0120	↑**
Cadaverine	0.0005	0.0011	↑**	0.0175	0.0409	↑*
Valine	0.0002	0.0005	↑**			
Leucine	0.0013	0.0025	↑**			
5-Aminovalerate	0.0229	0.0253	↑*			
Uracil	0.0332	0.0349	↑*	0.0064	0.0224	↑**
Arginine	0.0003	0.0008	↑**			
Creatine	0.0002	0.0005	↑**	0.0347	0.0442	↑*
Malonate	0.0066	0.0093	↑**			
α-Ketoisocaproate	0.0080	0.0106	↑**			
Methylamine	0.0005	0.0011	↑**			
2-Oxoglutarate	0.0022	0.0039	↓**	0.0479	0.0479	↑*
Threonine	0.0066	0.0098	↓**			
Lactate	0.0125	0.0154	↓*			
α-D-Xylp	0.0041	0.0066	↓**			
α-Arabinose	0.0341	0.0341	↓*			
Succinate	0.0148	0.0173	↓*			
Choline	0.0046	0.0074	↓**			
Pyruvate				< 0.0001	< 0.0001	↑**
Imidazole				0.0034	0.0160	↑**
Malate				0.0079	0.0222	↑*
Cytidine				0.0452	0.0487	↑*
Uridine				0.0357	0.0416	↑*
Taurine				0.0283	0.0396	↓*

*Abbreviations*: *α-D-Xylp* α-D-xylopyranosyl

†From Student t test. *p < 0.05; **p < 0.01. ‡Adjusted by false discovery rate method across the metabolites within each comparison. HFD vs CD: compare HFD to CD; LF vs HFD: compare LF to HFD

**Fig. 5:**
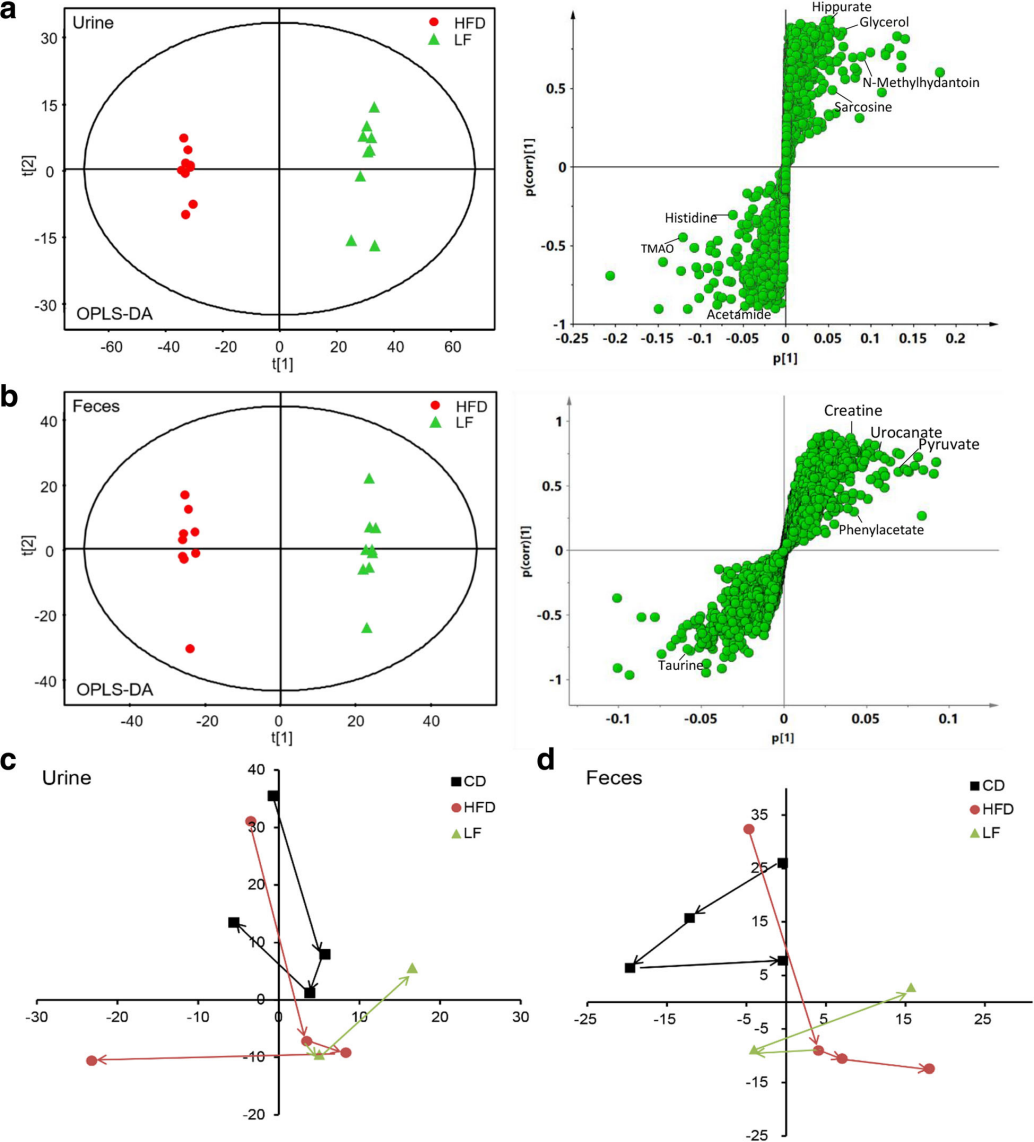
**a** Urine OPLS-DA score plots and S-plots of the HFD and LF group based on ^1^H NMR technology (HFD vs. CD). **b** Feces OPLS-DA score plots and S-plots of the HFD and LF group based on ^1^H NMR technology (LF vs. HFD). Some makers were labeled in the S-plots. PCA mean score trajectory plots on pretreatment day − 1 and at weeks 4, 8, and 12 in urine (**c**) and feces (**d**), respectively

The effects of LF combined with the high fat diet on the metabolic profiles of urine and feces were also investigated using OPLS-DA (Fig. 5). In urine, LF intervention caused significant elevations in the levels of *N*-methyl-4-pyridone-3-carboxamide (4-PY), hippurate, ethanol, *N,N*-dimethylglycine, glycerol, *N*-methylhydantoin, sarcosine and xanthine together with decreased levels of acetamide, histidine, pyruvate, formate and trimethylamine *N*-oxide (Fig. 5, Table 1). Furthermore, in fecal extracts, LF treatment led to higher levels of fecal pyruvate, imidazole, uracil, tyrosine, urocanate, 3-phenylpropionate, phenylacetate, malate, 2-oxoglutarate, cytidine, uridine, creatine and cadaverine but a lower level of taurine compared with HFD feeding.

Metabolomics analysis of fecal and urine suggested that the metabolic profiles of HFD rats deviated from those of CD rats. After the LF intervention, we found that the metabolic profiles transformed in a different way compared with HFD control rats. We analyzed the HFD-induced and LF-induced metabolic alterations at four time points to characterize the time-dependent endogenous metabolic response in urine and feces (Fig. 5c, d). The results showed that the metabolic trajectories of rats diverged from their initial metabolic position with the occurrence of HFD-induced obesity, and that the metabolic trajectories of the LF group were closer to those of the CD group, suggesting that LF-induced changes in metabolic pathways may be related to treating obesity.

### Perturbed metabolic pathways in response to HFD and LF

**Fig. 6:**
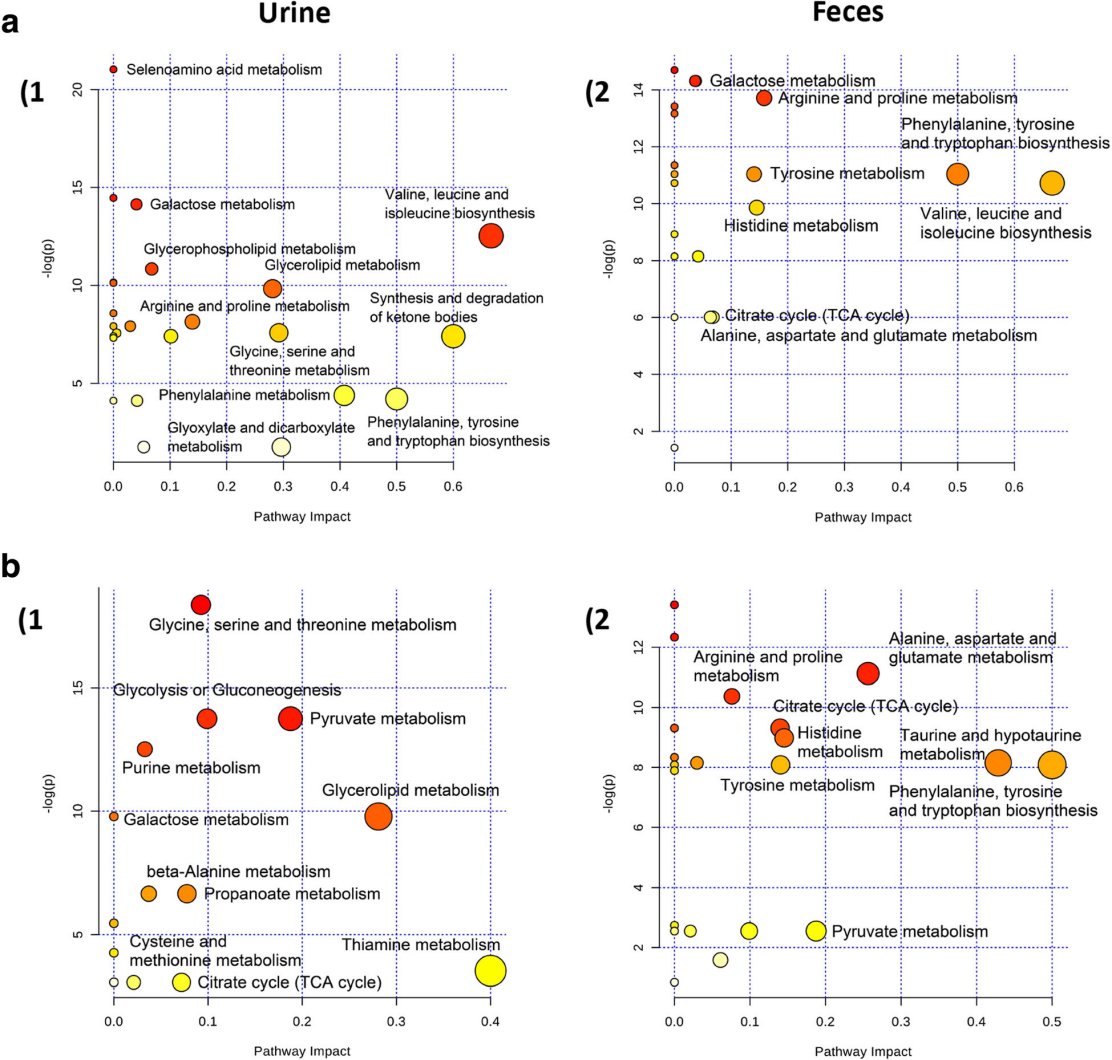
Meaningful metabolic pathways of urine and feces. Metabolism pathways in the urine and feces of HFD rats compared with CD or LF rats were visualized by bubble plots. Bubble size is proportional to the impact of each pathway and bubble color denotes the significance from highest in red to lowest in white. (**a**-1) HFD vs CD, metabolism pathways associated with urinary metabolites; (**a**-2) HFD vs CD, metabolism pathways associated with fecal metabolites; (**b**-1) LF vs HFD, metabolism pathways associated with urinary metabolites; (**b**-2) LF vs HFD, metabolism pathways associated with fecal metabolites

**Fig. 7:**
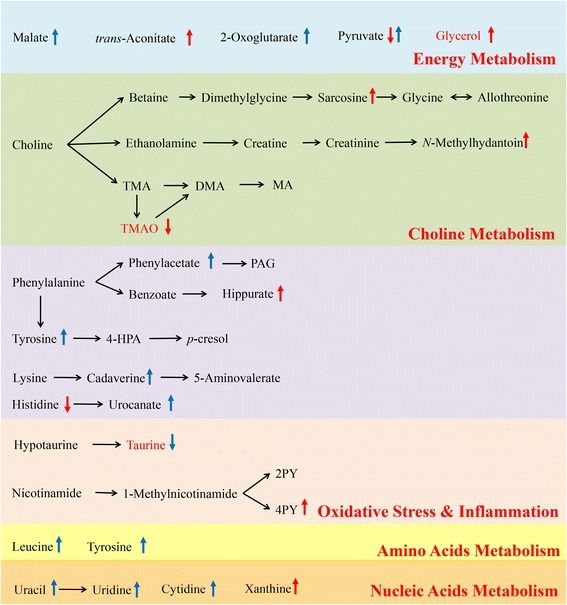
Metabolic pathways altered by LF intervention (compared with HFD control group). ↑, up-regulated; ↓, down-regulated; red color: urine; blue color: feces. Abbreviations: TMA, Trimethylamine; DMA, Dimethylamine; MA, Methylamine; TMAO, Trimethylamine N-oxide; PAG, Phenylacetylglycine; 2PY, N′-methyl-2-pyridone-5-carboxamide; 4PY, N′-methyl-4-pyridone-3-carboxamide

To consider the most evident metabolic disturbances, MetPA was employed using a web-tool (http://www.metaboanalyst.ca) to identify significantly perturbed metabolic pathways in response to HFD and LF. Compared to CD rats, the significant perturbed metabolic pathways in the urine and feces of HFD-induced rats were determined (Fig. 6-A-1, −A-2). Five metabolic pathways were filtered out as the most important metabolic pathways (*p* < 0.05, impact> 0.1) related to urine metabolic disturbances, and nine were filtered out in feces, including valine, leucine and isoleucine biosynthesis, synthesis and degradation of ketone bodies, as well as phenylalanine, tyrosine and tryptophan biosynthesis. These metabolic alterations and associated pathways provided insights into the mechanisms involved in the development and progression of the model. These metabolic pathways were analyzed in the KEGG database (http://www.kegg.jp/), for further analysis and five meaningful metabolic pathways related to the model were summarized, including energy metabolism, choline metabolism, oxidative stress and inflammation, amino acid metabolism, and gut microbiota and host co-metabolism (Additional file 1: Figure S3). After the LF intervention, the significant perturbed metabolic pathways compared with HFD-induced obese rats in urine and feces were also determined (Fig. 6-B-1, -B-2). Three and five metabolic pathways were filtered out in urine and feces respectively, as the most important metabolic pathways (*p* < 0.05, impact> 0.1) related to metabolic disturbances. These metabolic pathways were analyzed in the KEGG database (http://www.genome.jp/kegg) for further analysis and six meaningful metabolic pathways related to the model are summarized in Fig. 7, including energy metabolism, choline metabolism, oxidative stress and inflammation, amino acid metabolism, gut microbiota and host co-metabolism, and nucleic acids metabolism.

## Discussion

A number of different prebiotics have recently been applied to treat of obesity and metabolic syndrome [3, 34]. LF, a mixture of lentinan and *Flos Lonicera* polysaccharide, is reported to have preventive and therapeutic effects on obesity and its complications, such as non-alcohol fatty liver disease (NAFLD) and diabetes [14, 35]. However, how LF works to prevent and treat obesity and its complications remains unknown. Therefore, in this study, we used a high-fat diet to induce obesity in SD rats and used LF for intervention. The data clearly indicated that a HFD-induced obese rat model was successfully established and a decrease in body weight gain and a reduction of fat accumulation occurred after the LF intervention (Fig. 1). A NMR-based metabolomics approach was applied to characterize the endogenous metabolic response to HFD and LF in urine and feces. The results showed that HFD disturbed the urine and fecal metabolic profiles and that LF reversed some of those changes. The key altered metabolites were widely distributed across energy metabolism, nucleic acid synthesis and the mammalian-microbial metabolic system as shown in Additional file 1: Figure S3 and Fig. 7.

### Energy metabolism

The tricarboxylic acid (TCA) cycle, the central hub connecting carbohydrate, fat and protein metabolism, is the main way that the body obtains energy and also provides raw materials for many biosynthesis processes in the body. The intake of a high-fat diet breaks the balance of energy intake and consumption and, disrupts the normal energy metabolism of the body, leading to obesity, fatty liver, insulin resistance and other diseases. Here, after a 12-week-HFD induction, the TCA cycle intermediates decreased significantly in the urine and feces of rats in the HFD group, including citrate, succinate, 2-oxoglutarate and fumarate. The results demonstrated that the TCA cycle is suppressed in an obese state, and this phenomenon is consistent with the previous literature [36]. As a comprehensive reflection of the energy metabolism level of the body, suppression of the TCA cycle indicates that the level of energy consumption decreased in an obese state, leading to energy surplus and fat accumulation. Compared with HFD rats, the metabolites of LF-treated rats presented an up-regulation in TCA cycle intermediates, such as malate and 2-oxoglutarate. These results revealed that LF can ameliorate the body’s obesity state by affecting energy metabolism pathway.

Reduction of the TCA cycle could lead to metabolic disturbance and obesity in the body, but how a HFD reduces the level of the TCA cycle is unknown. According to the disturbed metabolic pathways (Fig. 7, Additional file 1: Figure S3), we demonstrated that a higher intake of fatty acids in HFD rats resulted in an up-regulated synthesis of liver fat. Glycerol, as well as fatty acids, is an ingredient in fat synthesis in the liver, which meets the increased demand along with increased synthesis of liver fat. The increased demand of glycerol results in a larger proportion of glucose being used for the synthesis of glycerol and fat to store energy in the HFD group, leading to competitive inhibition of the conversion of glucose into pyruvate and energy. Thus, TCA cycle intermediates decreased in an obese state, as well as a reduction of glycerate-3-phosphate and glycerol due to their transformation into fat. This finding also confirmed the experimental phenomena of the increase of epididymal fat weight and liver lipid accumulation in HFD group rats. Interestingly, the LF treatment up-regulated the levels of glycerol and TCA cycle intermediates, and ameliorated energy metabolic disorders in obese individuals.

### Choline metabolism and gut microbiota metabolism

Although choline can be synthesized endogenously in the human body, choline mainly comes from food, such as egg yolks, animal liver, fish, nuts, beans and so on, and choline is one of the necessary nutrients for body growth and development [37]. Most dietary choline is digested and absorbed in the small intestine, and can then be phosphorylated through intestinal mucosal cells to form phosphate choline. Choline can also be oxidized to betaine in the liver or kidney, and can also form acetylcholine in the nervous system [38]. Unabsorbed choline can be transformed into trimethylamine (TMA) by anaerobic bacteria after entering the large intestine. TMA can be further decomposed into dimethylamine (DMA) and methylamine (MA), and can also be transported into the liver and oxidized to trimethylamine oxide (TMAO) [39]. Studies have shown that TMAO can promote the formation of arterial plaques, which are associated with the occurrence and development of cardiovascular disease [40]. Additionally, it was reported that intestinal flora can influence the formation and development of atherosclerosis [41]. TMAO is a direct risk factor for atherosclerosis, and the content of TMAO, the metabolite of choline in food, depends on the metabolism of intestinal flora [42]. Gut microbiota serve as a filter for our largest environmental exposure--what we eat. They also function, fundamentally, as a dynamic endocrine organ, generating biologically active metabolites in response to specific nutrient inputs that can enter the circulation and elicit biological effects at distant sites within the host [43]. The metabolic products of intestinal flora, such as short chain fatty acids (SCFAs), polyphenols, and methylamine, have an important effect on atherosclerosis. In this study, after high-fat diet induction, the choline content decreased significantly in the feces of obese rats, indicating that a HFD may promote the growth of intestinal bacteria that can break down choline. Gut choline was extensively broken down into TMA, and some of the TMA was oxidized to TMAO within the liver, which could be transformed into DMA by a demethylation enzyme. These metabolites are excreted in the urine, causing a rise of the DMA and TMA contents in the urine of HFD rats. These results revealed that HFD caused disorders in choline metabolism, accelerated the decomposition of choline and reduced the activity of choline. As choline can promote the decomposition of adipose tissue, the decrease of the choline content can lead to an accumulation of fat in the liver and may eventually induce non-alcoholic fatty liver [44].

Polysaccharides is a major nutrient source for intestinal flora, and some polysaccharides can be converted into digestible substances such as monosaccharides and short chain carboxylic acids, providing energy for bacteria or hosts [45]. Here, we found a reduction of TMAO in the urine of LF-treated rats, suggesting that LF could have a potential role in reducing risk of atherosclerosis in obese rats. We suspect that not only can intestinal bacteria digest polysaccharides, but that polysaccharides can also influence the structure of intestinal flora in return, therefore reducing the incidence of cardiovascular disease.

### Oxidative stress and inflammation

In addition to choline metabolism, we also observed that nicotinic acid metabolism was disturbed in the HFD group. The bioactive form of nicotinic acid (vitamin B3) is nicotinamide, which is an important coenzyme in the body. Nicotinic acid metabolism can affect choline metabolism through betaine and glycine metabolism [46]. There are two main sources of nicotinamide: one is from food, and the other is the transformation of tryptophan in the liver. Nicotinamide is converted to oxidized nicotinamide or 1-methylnicotinamide by liver-related enzymes, and 1-methylnicotinamide can be further oxidized into N′-methyl-2-pyridone-5-carboxamide (2PY) and N′-methyl-4-pyridone-3-carboxamide (4PY) [47]. In this study, the content of tryptophan, nicotinamide and 2PY in the urine of rats in the HFD group increased significantly, indicating that a high-fat diet promoted the metabolism of nicotinamide. Nicotinamide is the precursor of nicotinamide adenine dinucleotide (NAD^+^) and nicotinamide adenine dinucleotide phosphate (NADP^+^) in many biochemical reactions in the body, and these coenzymes transmit electrons and protons in the oxidation-reduction reactions of the body, promoting antioxidation effects. The results showed that, in response to oxidative stress induced by a high-fat diet, the body enhanced the metabolism of nicotinamide to exhibit antioxidant activities. Taurine also has antioxidant properties, related to the oxidative stress level [48], and elevated taurine in the urine indicates a rising level of taurine in the body, which may play an antioxidant role to relieve the oxidative stress produced by a high-fat diet. In addition, taurine can be combined with bile acid to form taurocholate to increase the solubility of lipids and cholesterol, relieve bile obstructions and inhibit the formation of cholesterol stones. In this experiment the HFD induced a persistently marked increase in the content of taurine in rats. This positive feedback regulation demonstrated that the body accelerated the synthesis of taurine to break down the excess fat from food in response to the high-fat intake. However, obesity occurred in HFD rats, which may indicate that the accumulation of fat was much faster than the hydrolysis of fat by taurine or due to other complicated reasons that contributed to fat metabolism. In the LF group, the content of taurine decreased, indicating an improvement of the body’s oxidative stress and inflammation compared with the HFD group.

## Conclusions

According to weight and various biochemical indexes, we successfully established a rat obesity model by 8 weeks of high-fat diet feeding in which the obesity status was improved after LF treatment. The results of the histopathology, biochemical indices and metabolomics suggested that LF improvement on obesity and its complications. The variations in urinary and fecal metabolites were clarified by a combination of a NMR metabolomics study and a multivariable statistical analysis. We found that the HFD affected energy metabolism, lipid metabolism, inflammatory response and the oxidative stress response in rats and that some of the metabolic disorders were ameliorated by LF. Considering the LF effect on body weight and metabolic profiling, it may be a potential weight-loss agent. Advances in technology in metabolic phenotyping methods have improved our ability to estimate and explore the pharmacology and mechanism of natural drugs in a dynamic and non-invasive manner.

## Additional file

Additional file 1: **Figure S1.** Effects of LF on body weight gain, liver weight, epididymal adipose weight and mean energy take in HFD-fed rats. (A) Body weight gain in 12 weeks; (B) liver weight; (C) epididymal adipose weight; (D) mean energy intake. Values are expressed as means ± standard error. Graph bars with different letters on top represent statistically significant results (*p* < 0.05) based on one-way ANOVA analysis, whereas bars with the same letter correspond to results that show no statistically significant differences. In the case where two letters are present on top of the bars in A, C, D, each letter should be compared separately with the letters of other bars to determine whether the results show statistically significant differences. **Figure S2.** PCA scores plots of ^1^H NMR data of urine (A) and feces (B) are exhibited at 12th week. Both analysis among three groups and pairwise comparisons are showed with PCA model. **Figure S3.** Metabolic pathways altered by HFD (compared with CD group). ↑, up-regulated; ↓, down-regulated; red color: urine; blue color: feces. Abbreviations: TMA, Trimethylamine; DMA, Dimethylamine; MA, Methylamine; TMAO, Trimethylamine N-oxide; PAG, Phenylacetylglycine; TCA cycle, Tricarboxylic acid cycle; 2PY, N′-methyl-2-pyridone-5-carboxamide; 4PY, N′-methyl-4-pyridone-3-carboxamide; NAD^+^, nicotinamide adenine dinucleotide; NADP^+^, nicotinamide adenine dinucleotide phosphate; 4-HPA, 4-hydroxybenzoacetic acid; NAG, N-acetyl-beta-D-glucosaminidase. **Table S1.** Recipes of control and high-fat diet. **Table S2.** ^1^H NMR data for metabolites in rat urine and feces. **Table S3.** Model validation parameters for rat urine. **Table S4.** Model validation parameters for rat feces. **Table S5.** Model validation parameters from confusion matrix. **Table S6.** Content changes of significant altered metabolites in urine. **Table S7.** Content changes of significant altered metabolites in feces. (DOCX 1259 kb)

## Abbreviations

Alt: Alanine aminotransferase
ANOVA: Analysis of variance
Ast: Aspartate aminotransferase
CD: Control diet
FLP: *Flos Lonicera* polysaccharides
Glu: Serum glucose
HDL-C: High-density lipoprotein cholesterol
HFD: High-fat diet
HMBC: Heteronuclear Multiple Bond Correlation spectroscopy
HMDB: Human Metabolome Data Base
HSQC: ^1^H-^13^C Heteronuclear Single Quantum Correlation
IACUC: Institutional Animal Care and Use Committee
LDL-C: Low-density lipoprotein cholesterol
LF: Lentinan and *Flos Lonicera* polysaccharides
MetPA: Metabolomics Pathway Analysis
NAFLD: Non-alcohol fatty liver disease
NMR: Nuclear magnetic resonance
OPLS-DA: Orthogonal projection to latent structure discriminant analysis
PCA: Principal Component Analysis
SD: Sprague-Dawley
SPF: Specific pathogen free
TC: Total serum cholesterol
TG: Triglycerides
TMAO: Trimethylamine oxide
TOCSY: ^1^H-^1^H Total Correlation Spectroscopy
UV: Unit variance
WHO: World Health Organization
