# Supplementary material for: Metabolomics insights into the modulatory effects of long-term compound polysaccharide intake in high-fat diet-induced obese rats
Source: Nutr Metab (Lond). 2018 Jan 23;15:8. doi: 10.1186/s12986-018-0246-2 (PMC5781284; doi:10.1186/s12986-018-0246-2)
Supplement: Additional file 1: — Figure S1. Effects of LF on body weight gain, liver weight, epididymal adipose weight and mean energy take in HFD-fed rats. (A) Body weight gain in 12 weeks; (B) liver weight; (C) epididymal adipose weight; (D) mean energy intake. Values are expressed as means ± standard error. Graph bars with different letters on top represent statistically significant results (p < 0.05) based on one-way ANOVA analysis, whereas bars with the same letter correspond to results that show no statistically significant differences. In the case where two letters are present on top of the bars in A, C, D, each letter should be compared separately with the letters of other bars to determine whether the results show statistically significant differences. Figure S2. PCA scores plots of 1H NMR data of urine (A) and feces (B) are exhibited at 12th week. Both analysis among three groups and pairwise comparisons are showed with PCA model. Figure S3. Metabolic pathways altered by HFD (compared with CD group). ↑, up-regulated; ↓, down-regulated; red color: urine; blue color: feces. Abbreviations: TMA, Trimethylamine; DMA, Dimethylamine; MA, Methylamine; TMAO, Trimethylamine N-oxide; PAG, Phenylacetylglycine; TCA cycle, Tricarboxylic acid cycle; 2PY, N′-methyl-2-pyridone-5-carboxamide; 4PY, N′-methyl-4-pyridone-3-carboxamide; NAD+, nicotinamide adenine dinucleotide; NADP+, nicotinamide adenine dinucleotide phosphate; 4-HPA, 4-hydroxybenzoacetic acid; NAG, N-acetyl-beta-D-glucosaminidase. Table S1. Recipes of control and high-fat diet. Table S2. 1H NMR data for metabolites in rat urine and feces. Table S3. Model validation parameters for rat urine. Table S4. Model validation parameters for rat feces. Table S5. Model validation parameters from confusion matrix. Table S6. Content changes of significant altered metabolites in urine. Table S7. Content changes of significant altered metabolites in feces. (DOCX 1259 kb) [file 12986_2018_246_MOESM1_ESM.docx]

**Supplementary Information**

Metabolomics Insights into the Modulatory Effects of Long-Term Compound Polysaccharide Intake in High-fat Diet Induced Obese Rats

Mingyi Chen^a^, Biyu Lu ^b^, Yuan Li^b^, Yuanyuan Wang^c^, Haihui Zheng^a^, Danmin Zhong^a^, Ziqiong Liao^a^, Mengxia Wang^b^, Fangli Ma^c^, Qiongfeng Liao*^b^ and Zhiyong Xie*^a^

*^a^School of Pharmaceutical Sciences, Sun Yat-sen University, Guangzhou, 510006, P. R. China*

*^b^School of Pharmaceutical Sciences, Guangzhou University of Chinese Medicine, Guangzhou, 510407, P. R. China*

*^c^ Infinitus (China) Company Ltd, Guangzhou, 510623, China.*

*Corresponding authors:

Zhiyong Xie

Tel./Fax: +86 20 39943047

E-mail address: xiezy2074@yahoo.com

Qiongfeng Liao

Tel./Fax: +86 20 3935 8081

E-mail: liaoqf2075@yahoo.com

**Figure S1.** Effects of LF on body weight gain, liver weight, epididymal adipose weight and mean energy take in HFD-fed rats. (A) Body weight gain in 12 weeks; (B) liver weight; (C) epididymal adipose weight; (D) mean energy intake. Values are expressed as means ± standard error. Graph bars with different letters on top represent statistically significant results (*p* < 0.05) based on one-way ANOVA analysis, whereas bars with the same letter correspond to results that show no statistically significant differences. In the case where two letters are present on top of the bars in A, C, D, each letter should be compared separately with the letters of other bars to determine whether the results show statistically significant differences.

**Figure S2.** PCA scores plots of ^1^H NMR data of urine (A) and feces (B) are exhibited at 12th week. Both analysis among three groups and pairwise comparisons are showed with PCA model.

**Figure S3.** Metabolic pathways altered by HFD (compared with CD group). ↑, up-regulated; ↓, down-regulated; red color: urine; blue color: feces. Abbreviations: TMA, Trimethylamine; DMA, Dimethylamine; MA, Methylamine; TMAO, Trimethylamine N-oxide; PAG, Phenylacetylglycine; TCA cycle, Tricarboxylic acid cycle; 2PY, N'-methyl-2-pyridone-5-carboxamide; 4PY, N'-methyl-4-pyridone-3-carboxamide; NAD^+^, nicotinamide adenine dinucleotide; NADP^+^, nicotinamide adenine dinucleotide phosphate; 4-HPA, 4-hydroxybenzoacetic acid; NAG, N-acetyl-beta-D-glucosaminidase.

**Table S1.** Recipes of control and high-fat diet

**Table S2.** ^1^H NMR data for metabolites in rat urine and feces

**Table S3.** Model validation parameters for rat urine

**Table S4.** Model validation parameters for rat feces

**Table S5.** Model validation parameters from confusion matrix

**Table S6** Content changes of significant altered metabolites in urine.

**Table S7** Content changes of significant altered metabolites in feces.


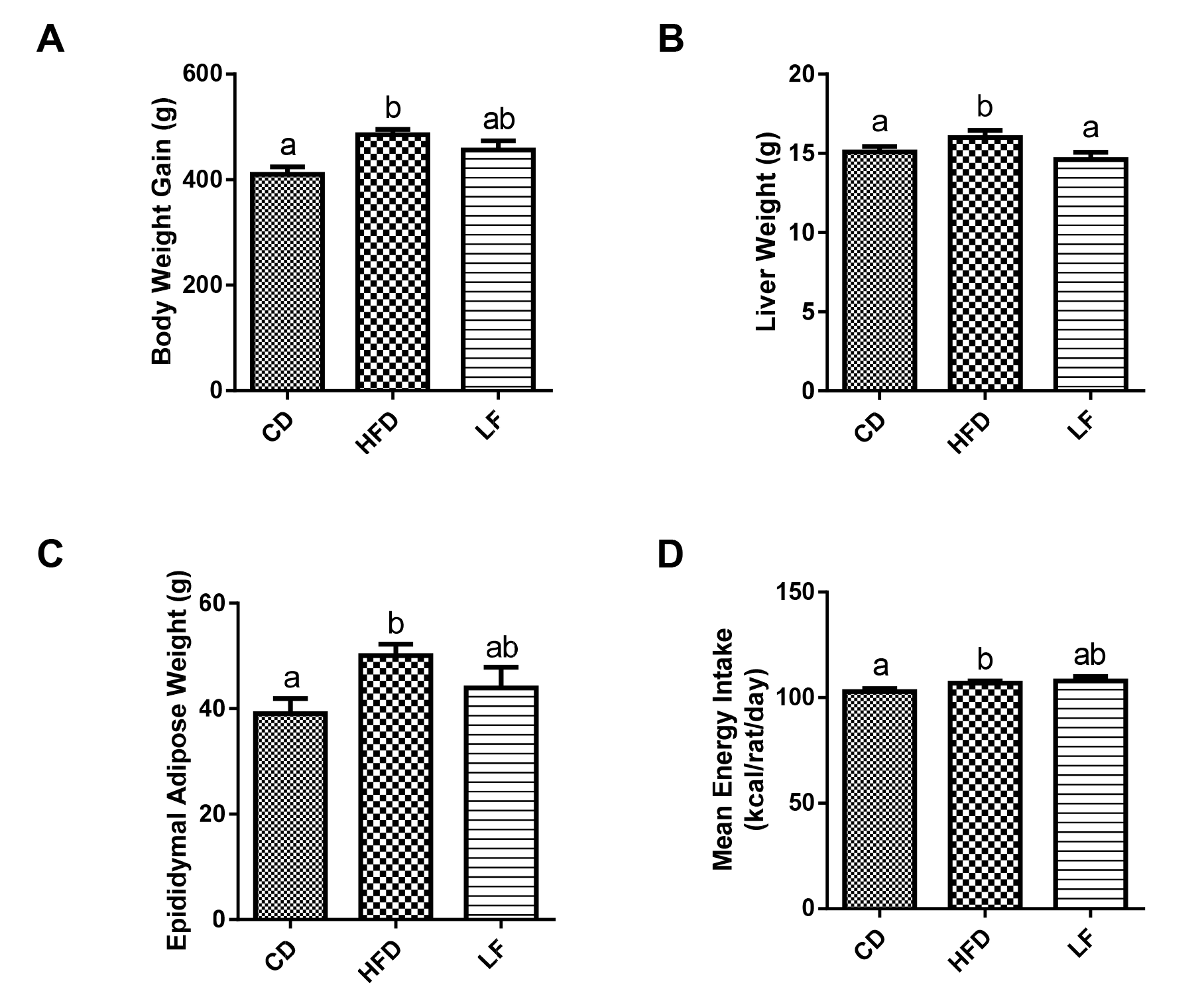


**Figure S1.** Effects of LF on body weight gain, liver weight, epididymal adipose weight and mean energy take in HFD-fed rats. (A) Body weight gain in 12 weeks; (B) liver weight; (C) epididymal adipose weight; (D) mean energy intake. Values are expressed as means ± standard error. Differences were assessed by ANOVA and denoted as follow: Values are statistically significant at * *p* < 0.05; a*, significantly different from CD group; b*, significantly different from HFD group (n=10).


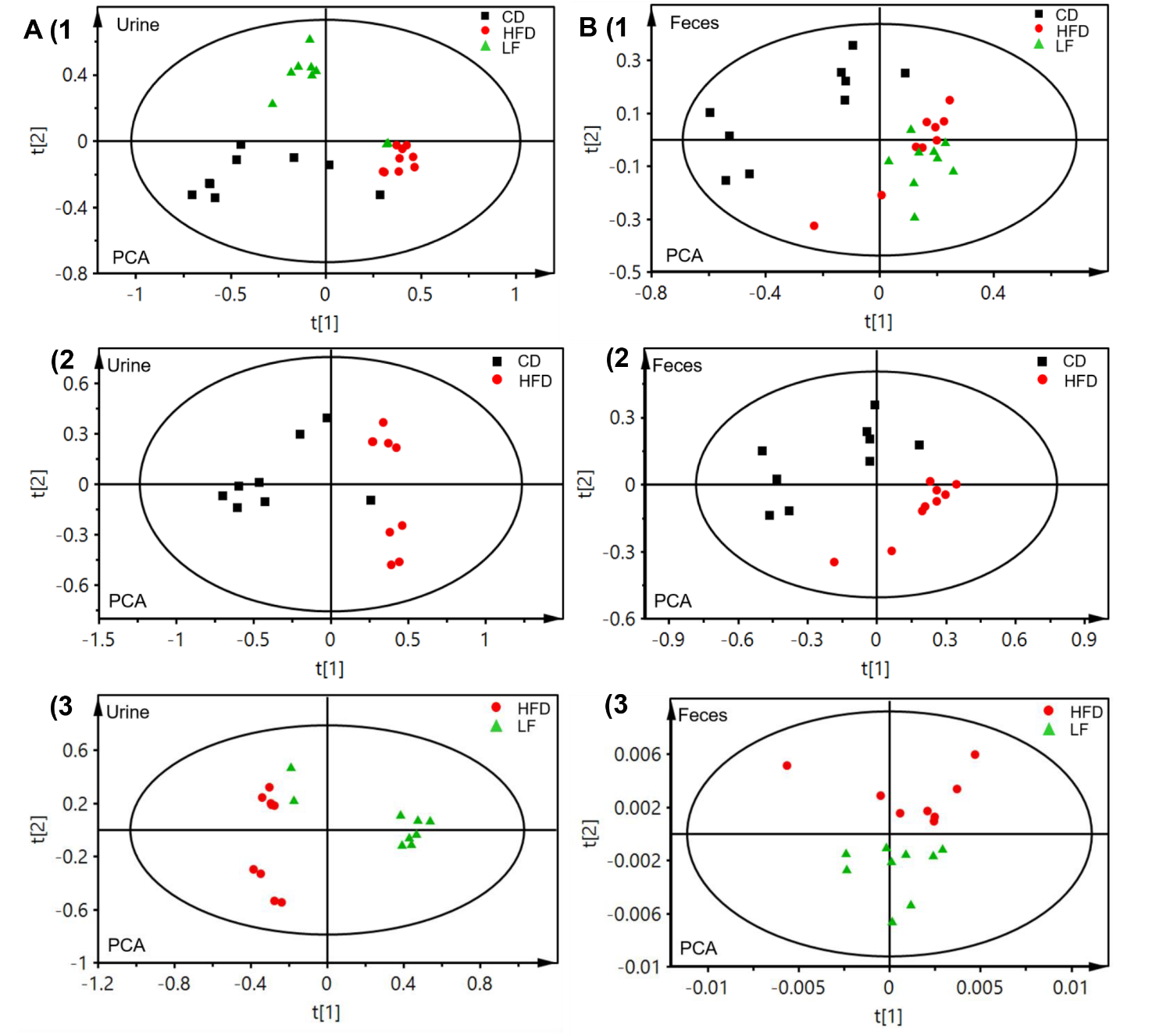


**Figure S2.** PCA scores plots of ^1^H NMR data of urine (A) and feces (B) are exhibited at 12 week. Both analysis among three groups and pairwise comparisons are showed with PCA model.


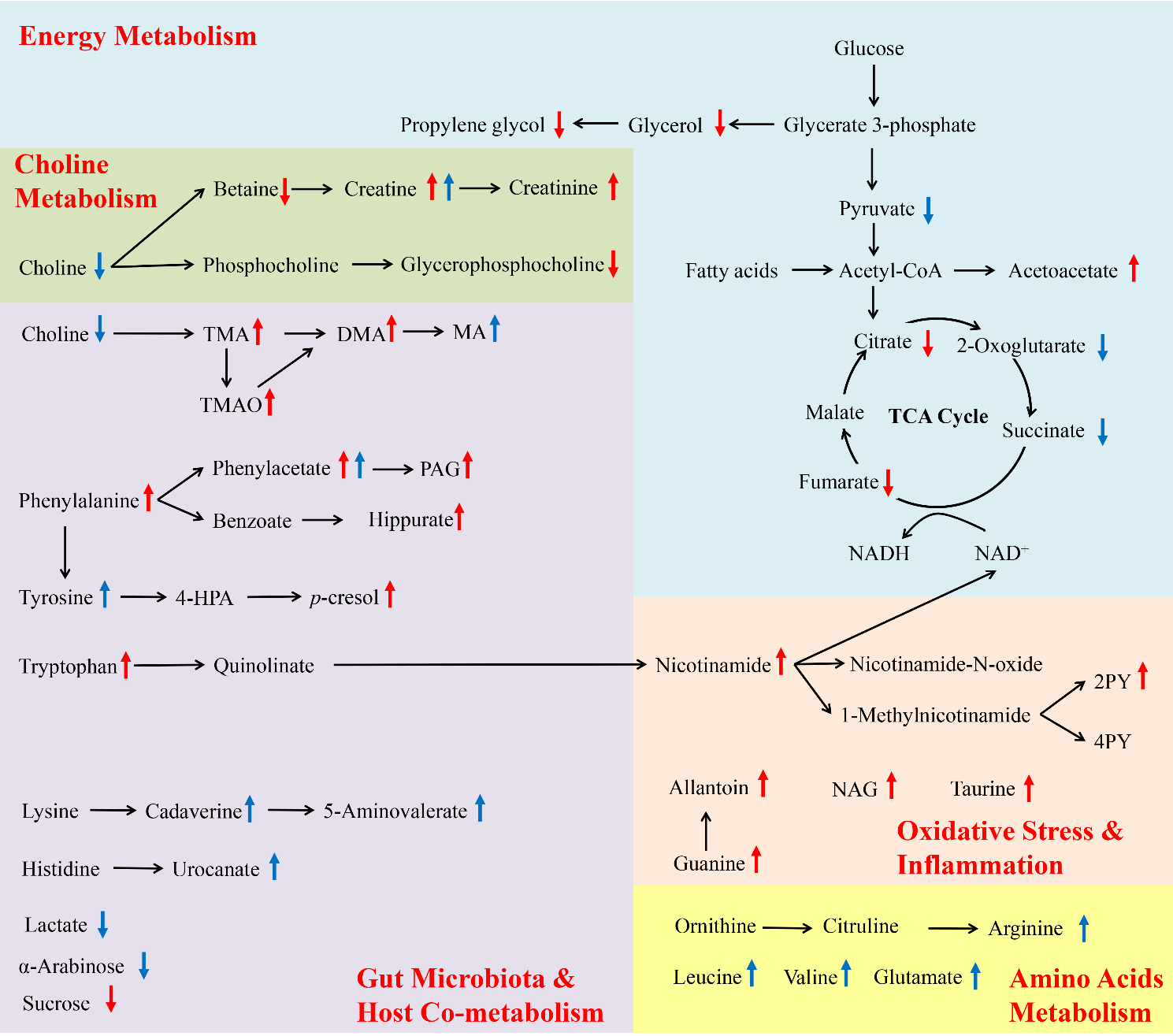
**Figure S3.** Metabolic pathways altered by HFD (compared with CD group). ↑, up-regulated; ↓, down-regulated; red color: urine; blue color: feces. Abbreviations: TMA, Trimethylamine; DMA, Dimethylamine; MA, Methylamine; TMAO, Trimethylamine N-oxide; PAG, Phenylacetylglycine.

TCA cycle, Tricarboxylic acid cycle; 2PY, N'-methyl-2-pyridone-5-carboxamide; 4PY, N'-methyl-4-pyridone-3-carboxamide; NAD^+^, nicotinamide adenine dinucleotide; NADP^+^, nicotinamide adenine dinucleotide phosphate; 4-HPA, 4-hydroxybenzoacetic acid; NAG, N-acetyl-beta-D-glucosaminidase.

**Table S1 Recipes of control and high-fat diet**

| Ingredient | Control diet (CD) | | High-fat diet (HFD) | |
| --- | --- | --- | --- | --- |
|  | g | kcal | g | kcal |
| Casein | 189.58 | 758.32 | 233.06 | 932.24 |
| L-Cystine | 2.84 | 11.36 | 3.50 | 14 |
| Com Starch | 298.59 | 1194.36 | 84.83 | 339.32 |
| Maltodextrin | 33.18 | 132.72 | 116.53 | 466.12 |
| Sucrose | 331.77 | 1327.08 | 201.36 | 805.44 |
| Cellulose | 47.40 | 0 | 58.26 | 0 |
| Soybean Oil | 23.70 | 213.30 | 29.13 | 262.17 |
| Lard | 18.96 | 170.64 | 206.84 | 1861.6 |
| Mineral Mix M1002 | 9.48 | 0 | 11.65 | 0 |
| Dicalcium Phosphate | 12.32 | 0 | 15.15 | 0 |
| Calcium Carbonate | 5.21 | 0 | 6.41 | 0 |
| Potassium Citrate | 15.64 | 0 | 19.23 | 0 |
| Vitamin Mix V1001 | 9.48 | 37.92 | 11.56 | 46.24 |
| Choline Bitartrate | 1.90 | 0 | 2.33 | 0 |
| Food Coloring | 0.047 | 0 | 0.058 | 0 |
| Total | 1000 | 3845.7 | 1000 | 4727.09 |

HFD+LF: HFD supplemented with a mixture of lentinan and *Flos Lonicera* polysaccharide (LF, 0.675%, *wt*/*wt*). More specifically, 1000 g HFD and 6.75 g LF were combined to obtain 1006.75 g HFD + LF.

**Table S2 ^1^H NMR data for metabolites in rat urine and feces**

| Key | Metabolites | δ ^1^H (ppm) | δ ^13^C | Samples^a^ |
| --- | --- | --- | --- | --- |
| 1 | Isocaproate | 0.88(d),1.45(m),1.48(d), 2.19(t) | —, 37.56, 30.7, 39.3 | F |
| 2 | 3-Methyl-2-oxovalerate | 0.89(t),1.09(d),1.44(m),1.66(m),2.93(m) | 13.2, 16.4, 26.8, 26.8, 45.9 | U |
| 3 | 2-Hydroxybutyrate | 0.89(t),1.69(m),1.73(m),3.97(dd) | 10.7, 29.5, —, 76.1 | U |
| 4 | Isovalerate | 0.90(d),1.93(m),2.05(d) | 24.4, 29.3, 49.3 | U |
| 5 | Valerate | 0.90(t),1.31(m),2.18(t) | 16.4, 23.9, 39.6 | F |
| 6 | Butyrate | 0.90(t),1.56(m),2.15(t) | 16.0, 22.1, 42.3 | F |
| 7 | Isovalerylglycine | 0.92(d),1.99(m),2.16(d),3.75(d) | 24.4, 30.2, 46.9, — | U |
| 8 | α-Ketoisocaproate | 0.92(d),2.06(m),2.61(d) | — | F |
| 9 | Isoleucine | 0.93(t),0.99(d),1.25(m),1.45(m),1.97(m),3.65(d) | 14.7, 17.2, 26.8, 26.8, 62.4 | U,F |
| 10 | Ketoleucine | 0.94(d),2.10(m),2.62(d) | 24.3, 26.2, — | U |
| 11 | Leucine | 0.96(d),0.97(d),1.69(m),1.71(m),3.74(m) | 23.4, 24.3, 26.6, 56.9 | U,F |
| 12 | Valine | 0.99(d),1.05(d),2.27(m),3.62(d) | 19.6, 20.5, 32.2, 63.4 | U,F |
| 13 | Isobutyrate | 1.05(d),2.38(m) | 22.1, 39.3 | U |
| 14 | Propionate | 1.06(t),2.19(q) | 12.9, 33.3 | F |
| 15 | 2-Methylglutarate | 1.07(d),1.60(m),1.75(m),2.15(t),2.24(m) | — | U |
| 16 | α-Ketoisovalerate | 1.13(d),3.02(m) | 18.8, 39.5 | F |
| 17 | Propylene glycol | 1.15(d),3.44(dd),3.56(dd),  3.88(m) | — | U |
| 18 | Isopropanol | 1.15(d),4.02(m) | 26.1, — | U |
| 19 | Allothreonine | 1.19(d),4.26(m) | 22.0,70.2 | U |
| 20 | Ethanol | 1.19(t),3.65(q) | 19.5, — | U |
| 21 | Methylmalonate | 1.25(d),3.17(q) | 18.0,54.7 | U |
| 22 | Fucose | 1.25(d),3.77(m),3.81(m),5.21(d) | 17.8,72.8,72.8,95.6 | U |
| 23 | 3-Hydroxyisovalerate | 1.27(s),2.38(s) | 30.2,51.6 | U |
| 24 | Threonine | 1.33(d),3.60(d),4.26(m) | 23.5, 63.2, 68.2 | U,F |
| 25 | Lactate | 1.34(d),4.11(q) | 22.9, 71.4 | U,F |
| 26 | 2-Hydroxyisobutyrate | 1.36(s) | 30.0 | U |
| 27 | Lysine | 1.42(m),1.73(m),1.87(m),3.02(t),  3.74(t) | 23.4, 29.7, —, 41.5, 57.6 | U |
| 28 | Cadaverine | 1.48(d),1.72(m),3.02(t) | 25.9, 29.4, 41.4 | F |
| 29 | Alanine | 1.49(d),3.79(q) | 18.3, 53.3 | U,F |
| 30 | Citrulline | 1.56(m),1.86(m),3.12(d),3.74(t) | —, —, 42.0, 57.9 | U,F |
| 31 | 5-Aminovalerate | 1.62(m),1.65(m),2.24(t),3.02(t) | 25.6, 29.7, 39.3, 41.4 | F |
| 32 | Arginine | 1.73(m),1.93(m),3.23(t),3.75(t) | 30.5,30.5,44.6,57.5 | F |
| 33 | Ornithine | 1.77(m),1.83(m),1.92(m) ,3.04(t),3.78(t) | 25.5, 25.5, —, 41.1, 56.8 | U |
| 34 | N-Acetylglutamate | 1.83(m),2.04(s),2.27(t),4.14(t) | — | U |
| 35 | Acetate | 1.93(s) | 26.5 | U,F |
| 36 | Acetamide | 1.99(s) | 25.0 | U |
| 37 | Proline | 2.01(m),2.07(m),2.36(m),3.34(m),3.45(m),4.13(m) | 25.8, 32.1, 48.7, 48.7, 63.5 | F |
| 38 | N-Acetylglycine | 2.03(s),3.76(d),7.98(s) | 24.2, 46.1, — | U |
| 39 | NAG | 2.04(s) | 23.2 | U |
| 40 | Glutamate | 2.06(m),2.10(m),2.36(m),3.75(m) | — | F |
| 41 | Methionine | 2.14(s),2.20(m),2.65(t),3.86(m) | 16.0, 31.3, 29.4,56.7 | F |
| 42 | O-Acetylcholine | 2.14(s),3.20(s),3.72(m),4.54(m) | 23.4, 56.8, 67.1, 60.2 | U |
| 43 | Levulinate | 2.21(s),2.39(t),2.77(t) | 32.4, 34.7, 41.1 | U |
| 44 | Acetone | 2.23(s) | 28.2 | U |
| 45 | p-cresol | 2.25(s),6.82(d),7.13(d) | 22.1,117.9 | U |
| 46 | Acetoacetate | 2.27(s),3.44(s) | 32.3, — | U |
| 47 | p-Cresol glucuronide | 2.30(s),7.05(s),7.23(d) | 22.3, 123.2, 135.6 | U |
| 48 | Malate | 2.38(dd),2.67(dd),4.31(dd) | 45.5,45.5, 73.5 | F |
| 49 | Pyruvate | 2.38(s) | 29.5 | U,F |
| 50 | Succinate | 2.41(s) | 37.3 | U,F |
| 51 | Carnitine | 2.41(t),3.23(s),3.41(m),4.55(m) | —, 57.5, 71.6, 66.4 | U |
| 52 | Thymidine | 2.44(m),4.44(dd),6.27(t),7.68(s) | 40.3,74.0,89.1,145.2 | U |
| 53 | 4-Pyridoxate | 2.44(s),4.76(s),7.85(s) | 20.4, — ,138.8 | U |
| 54 | 2-Oxoglutarate | 2.44(t),3.01(t) | 33.2, 39.0 | U,F |
| 55 | Thiamine | 2.48(s),2.56(s),3.18(t),3.89(t), 5.45(s),8.04(s) | 27.1,13.9,31.9,63.9,54.2,160.0 | U |
| 56 | 3-Phenylpropionate | 2.49(t),2.88(d),7.27(t),7.30(d), 7.37(m) | —, 34.4, —, —, 131.2 | F |
| 57 | Citrate | 2.55(d),2.69(d) | 48.7, 48.7 | U |
| 58 | Methylamine | 2.61(s) | 27.0 | U,F |
| 59 | Hypotaurine | 2.66(t),3.37(t) | 58.5,37.0 | U |
| 60 | Aspartate | 2.67(dd),2.82(dd),3.91(dd) | 39.3, 39.3, 55.1 | F |
| 61 | Dimethylamine | 2.72(s) | 36.8 | U,F |
| 62 | Sarcosine | 2.76(s),3.65(s) | 34.8, 53.7 | U |
| 63 | Succinimide | 2.78(s) | 41.9 | U |
| 64 | Trimethylamine | 2.87(s) | 47.4 | U,F |
| 65 | N,N-Dimethylglycine | 2.93(s),3.72(s) | 46.1, 62.2 | U |
| 66 | Creatine | 3.05(s),3.93(s) | 40.1, 56.2 | U,F |
| 67 | Creatinine | 3.05(s),4.05(s) | 33.5, 58.4 | U |
| 68 | Tyrosine | 3.06(d),3.15(dd),3.94(dd),6.90(d),7.20(d) | 37.9, 37.8, 59.4, 118.8, 133.4 | F |
| 69 | τ-Methylhistidine | 3.06(dd),3.16(dd),3.68(s),3.96(d),7.00(s),7.66(s) | 30.7, 36.5, 58.1, 122.9, — | U |
| 70 | Malonate | 3.11(s) | 50.1 | F |
| 71 | cis-Aconitate | 3.12(d),5.72(t) | 46.7, 127.0 | U |
| 72 | Phenylalanine | 3.12(dd),3.27(dd),4.00(q),7.32(d),7.36(t),7.42(m) | 38.9, 38.9, 58.9, 132.2, 129.8, 131.9 | U |
| 73 | Histidine | 3.14(dd),3.25(dd),4.01(d),7.09(s),7.83(s) | —, —, —, 119, 139.1 | U,F |
| 74 | Dimethyl sulfone | 3.14(s) | — | U |
| 75 | Ethanolamine | 3.14(t),3.83(t) | 45.4, 60.2 | F |
| 76 | N-Nitrosodimethylamine | 3.16(s),3.80(s) | 32.2, 39.8 | U |
| 77 | Choline | 3.20(s),3.52(m),4.07(m) | 56.9, 70.1, 58.2 | U,F |
| 78 | Phosphorylcholine | 3.20(s),3.67(t),4.19(dd) | 56.0, 68.5, 60.3 | U |
| 79 | Glycerophosphocholine | 3.23(s),3.69(m),3.92(m),4.30(m) | 55.9, 68.1, 73.9, 64.4 | U |
| 80 | β-Glucose | 3.24(dd), 3.41(t), 3.46(m), 3.49(t), 3.77(dd), 3.90(dd), 4.65(d) | —, —, —, 63.2, 63.2, 98.4 | U,F |
| 81 | Betaine | 3.25(s),3.89(s) | 57.0, 68.0 | U |
| 82 | Trimethylamine-N-oxide | 3.26(s) | 61.5 | U |
| 83 | Taurine | 3.27(t),3.43(t) | 49.5, 37.5 | U,F |
| 84 | Lactose | 3.27(t),3.51(m),3.60(m),3.71(m), 3.77(m),3.85(m),3.94(m),4.45(d), 4.66(d), 5.21(d) | 76.2, —, —, —, 64.6, 63.5, —, 104.6, 98.9, 93.9 | U |
| 85 | Tryptophan | 3.31(dd),3.49(dd),4.06(dd), 7.21(m),7.29(m),7.33(s),7.54(d), 7.74(d) | — | F |
| 86 | Methanol | 3.37(s) | 51.2 | F |
| 87 | trans-Aconitate | 3.43(s),6.59(s) | 39.2, 133.4 | U |
| 88 | α-Glucose | 3.43(t),3.48(m),3.53(dd),3.71(t), 3.73(dd),3.83(m), 3.85(dd), 5.24(d) | 72.4, 74.3, 75.4, —, —, 75.2, 94.9 | U,F |
| 89 | 4-Hydroxyphenylacetate | 3.44(s),6.86(d),7.16(d) | 46.9,118.2,133.4 | U |
| 90 | Sucrose | 3.49(d),3.56(dd),3.74(dd), 3.85(m),4.06(d),4.22(d),5.42(d) | 72.2,75.5, —, 75.3,76.9,79.3,95.3 | U |
| 91 | Phenylacetate | 3.53(s),7.27(d),7.29(s),7.36(t) | 47.2,129.6, 131.1 | U |
| 92 | Glycerol | 3.55(t),3.63(m),3.77(m) | — | U |
| 93 | Glucuronate | 3.57(d),3.72(m),4.07(d),4.64(d), 5.24(d) | — | U |
| 94 | Glycine | 3.57(s) | 44.5 | U,F |
| 95 | Glycogen | 3.63(m),3.66(m),3.83(dd), 3.87(dd),3.97(d),5.39(s) | 72.6, 79.6, 72.5, 63.2, 101.8 | U |
| 96 | Galactonate | 3.64(dd),3.70(d),3.72(s),3.97(m), 4.26(d) | — | U |
| 97 | 2PY | 3.65(s),6.67(d),7.96(dd),8.34(d) | 41.4,121.9,142.3,145.5 | U |
| 98 | Mannitol | 3.67(dd); 3.77(m); 3.81(d); 3.87(dd) | 65.4, 72.8, 72.6, 66.7 | U |
| 99 | Phenylacetylglycine | 3.67(s),3.74(d),7.35(t),7.41(t) | 44.4, —, 119.3,131.1 | U |
| 100 | Indole-3-acetate | 3.68(s),7.16(t),7.21(t),7.50(d), 7.63(d) | 36.6, 121.2, 126.5, 114.4, 121.3 | U |
| 101 | Guanidoacetate | 3.80(s) | 47.5 | U |
| 102 | Uridine | 3.81(d),3.92(d),4.24(t),4.36(t), 5.90(d),5.91(d),7.87(d) | —,64.0,69.0,77.3,105.5,92.8,145.4 | F |
| 103 | α-Galactose | 3.81(dd),3.85(dd),3.99(m), 4.09(m),5.27(d) | 71.7,73.3,72.5, —, 95.3 | F |
| 104 | Glycerate 3-phosphate | 3.84(d),4.06(d),4.22(m) | 64.5, —, 80.0 | U |
| 105 | α-Arabinose | 3.85(dd),3.90(m),3.99(t),4.03(dd),5.24(d) | —, —, 72.3, 66.3, 93.9 | F |
| 106 | Inosine | 3.85(dd),3.92(dd),4.28(q),4.44(t),6.11(d),8.24(s),8.34(s) | 63.8, —, 88.9,73.7,91.4,149.8,143.6 | F |
| 107 | Glycylproline | 3.88(d),3.94(s) | 49.0,42.7 | U |
| 108 | Glycolate | 3.94(s) | 64.2 | U |
| 109 | Hippurate | 3.96(d),7.55(t),7.64(t),7.83(dd), 8.52(s) | 46.1, 131.5, 134.9, 129.9 | U |
| 110 | 4-Hydroxyhippurate | 3.97(s),6.97(d), 7.76(d) | — | U |
| 111 | β-Ribofuranose | 4.00(m),5.26(d) | 78.3,103.9 | U |
| 112 | Pseudouridine | 4.01(dd),4.03(dd),4.05(dd), 4.16(d),4.29(dd),4.69(d),7.68(s) | 64.4,64.4,74.1,73.5,76.4,81.9,144.5 | U |
| 113 | Cytidine | 4.13(m),4.24(t),5.91(d),6.07(d), 7.85(d) | — | F |
| 114 | Tartrate | 4.35(s) | 77.4 | U |
| 115 | 1-Methylnicotinamide | 4.48(s),8.18(t),8.90(d),8.96(d), 9.27(s) | 50.8, 131.1, —, —, — | U |
| 116 | Allantoin | 5.39(s),6.02(s),7.27(s),8.01(s) | 66.5 | U |
| 117 | Uracil | 5.81(d),7.55(d) | 104.2, 146.2 | U,F |
| 118 | Cytosine | 5.98(d),7.51(d) | — | F |
| 119 | Urocanate | 6.40(d),7.30(d),7.36(s),7.86(s) | 133.4,124.7, 141.4, 134.2 | U,F |
| 120 | Fumarate | 6.52(s) | 137.5 | U,F |
| 121 | 4PY | 6.70(d),7.83(dd),8.55(d) | 123.4,146.8,149.2 | U |
| 122 | 4-Hydroxybenzoate | 6.90(d),7.82(d) | 117.2, 133.6 | U |
| 123 | Tryptamine | 7.20(td),7.27(td),7.50(d),7.70(d) | 123.3,126.1,115.3,120.7 | U |
| 124 | Imidazole | 7.33(s),8.30(s) | 123.2, 133.9 | F |
| 125 | Nicotinamide | 7.60(dd),8.27(dd),8.72(dd), 8.94(dd) | 127.0,139.0,154.2,150.1 | U |
| 126 | Guanine | 7.68(s) | 145.5 | U |
| 127 | Nicotinamide-N-oxide | 7.74(dd),8.12(m),8.49(m),8.75(t) | 130.2,133.4,144.2,141.4 | U |
| 128 | Xanthine | 7.87(s) | 141.8 | U |
| 129 | Adenine | 8.19(s),8.21(s) | 144.2, 156.2 | F |
| 130 | Oxypurinol | 8.21(s) | 129.1 | U |
| 131 | Formate | 8.46(s) | 172.0 | U,F |

^a^ Corresponding sample of metabolites. U = Urine; F = Feces; MOVA = 3-Methyl-2-oxovalerate; NAG = N-acetyl-beta-D-glucosaminidase; NDMA = Nitrosodimethylamine; GPC = Glycerophosphocholine; TMAO = Trimethylamine-N-oxide; 4-HPA = 4-Hydroxyphenylacetate; 2PY = N-methyl-2-pyridone-5-carboxamide; 4PY = N-methyl-4-pyridone-3-carboxamide.

**Table S3 Model validation parameters for rat urine**

| Urine | CD vs. HFD | | HFD vs. LF | | CD vs. HFD vs. LF | | |
| --- | --- | --- | --- | --- | --- | --- | --- |
|  | PCA | OPLS-DA | PCA | OPLS-DA | | PCA | OPLS-DA |
| R^2^X | 0.686 | 0.613 | 0.644 | 0.636 | | 0.692 | 0.676 |
| Q^2^ | 0.499 | 0.895 | 0.415 | 0.826 | | 0.515 | 0.834 |
| *p* | - | < 0.001 | - | 0.011 | | - | < 0.001 |

R^2^X represents the goodness of fit of models; Q^2^ represents the predictability of models; *p* was calculated by CV-ANOVA, and OPLS-DA was valid when *p* < 0.05.

**Table S4 Model validation parameters for rat feces**

| Feces | CD vs. HFD | | HFD vs. LF | | CD vs. HFD vs. LF | | |
| --- | --- | --- | --- | --- | --- | --- | --- |
|  | PCA | OPLS-DA | PCA | OPLS-DA | | PCA | OPLS-DA |
| R^2^X | 0.784 | 0.724 | 0.696 | 0.692 | | 0.817 | 0.794 |
| Q^2^ | 0.561 | 0.956 | 0.250 | 0.856 | | 0.601 | 0.880 |
| *p* | - | < 0.001 | - | < 0.001 | | - | < 0.001 |

R^2^X represents the goodness of fit of models; Q^2^ represents the predictability of models; *p* was calculated by CV-ANOVA, and OPLS-DA was valid when *p* < 0.05.

**Table S5 Model validation parameters from confusion matrix**

|  | CD vs. HFD | | HFD vs. LF | |
| --- | --- | --- | --- | --- |
|  | urine | feces | urine | feces |
| Sensitivity | 90.0% | 88.9% | 80.0% | 87.5% |
| Specificity | 90.0% | 87.5% | 90.0% | 100% |

**Table S6 Content changes of significant altered metabolites in urine.**

| Altered Metabolites | Fold Change | |
| --- | --- | --- |
|  | HFD/CD | LF/CD |
| Acetoacetate | 2.15 | 1.29 |
| Creatine | 1.54 | 1.41 |
| Creatinine | 1.43 | 1.30 |
| Allantoin | 1.40 | 1.29 |
| Phenylacetate | 1.34 | 1.27 |
| Hippurate | 1.31 | 1.42 |
| Phenylalanine | 1.33 | 1.26 |
| Succinimide | 1.30 | 1.25 |
| N-Acetylglutamate | 1.29 | 1.23 |
| Uracil | 1.24 | 1.19 |
| Valine | 1.22 | 1.17 |
| Levulinate | 1.21 | 1.16 |
| Alanine | 1.30 | 1.21 |
| 2-Methylglutarate | 1.27 | 1.24 |
| *p*-cresol | 1.17 | 1.13 |
| Leucine | 1.17 | 0.11 |
| Trimethylamine | 1.16 | 1.08 |
| 2-Hydroxybutyrate | 1.15 | 1.03 |
| Glycogen | 1.17 | 1.14 |
| N-Phenylacetylglycine | 1.14 | 1.10 |
| Dimethylamine | 1.73 | 1.19 |
| Carnitine | 1.07 | 1.01 |
| Taurine | 1.52 | 1.51 |
| Tryptophan | 1.13 | 1.11 |
| 2PY | 1.08 | 1.09 |
| Guanine | 1.22 | 1.18 |
| Nicotinamide | 1.32 | 1.27 |
| Histidine | 1.18 | 1.10 |
| Phosphorylcholine | 0.95 | 0.99 |
| Ornithine | 0.90 | 0.93 |
| N-Nitrosodimethylamine | 0.66 | 0.74 |
| Glycerol | 0.85 | 0.97 |
| Citrate | 0.79 | 0.94 |
| Betaine | 0.76 | 0.85 |
| Sucrose | 0.68 | 0.64 |
| Glycine | 0.59 | 0.61 |
| Glycerophosphocholine | 0.57 | 0.64 |
| Propylene glycol | 0.57 | 0.64 |
| Galactonate | 0.54 | 0.58 |
| Pseudouridine | 0.79 | 0.92 |
| Fumarate | 0.81 | 0.92 |
| 4PY | 0.89 | 1.21 |
| Ethanol | 0.90 | 1.01 |
| N-Methylhydantoin | 0.94 | 1.03 |
| Sarcosine | 0.90 | 1.02 |
| Xanthine | 0.91 | 0.99 |
| Acetamide | 1.11 | 1.05 |
| Pyruvate | 1.08 | 1.01 |
| Formate | 1.20 | 0.92 |
| Trimethylamine N-oxide | 1.11 | 0.64 |

Fold change with a value > 1 indicates a relatively lower concentration present in the rats from CD group in each cross-comparison. Abbreviations: 2PY, N'-methyl-2-pyridone-5-carboxamide; 4PY, N'-methyl-4-pyridone-3-carboxamide.

**Table S7 Content changes of significant altered metabolites in feces.**

| Altered Metabolites | Fold Change | |
| --- | --- | --- |
|  | HFD/CD | LF/CD |
| Imidazole | 1.33 | 1.79 |
| Urocanate | 1.28 | 1.43 |
| 3-Phenylpropionate | 1.28 | 1.42 |
| Glutamate | 1.25 | 1.31 |
| Phenylacetate | 1.24 | 1.35 |
| Tyrosine | 1.21 | 1.42 |
| Cadaverine | 1.19 | 1.26 |
| Valine | 1.18 | 1.05 |
| Leucine | 1.16 | 1.26 |
| 5-Aminovalerate | 1.16 | 1.22 |
| Uracil | 1.15 | 1.41 |
| Arginine | 1.13 | 1.13 |
| Creatine | 1.13 | 1.18 |
| Malonate | 1.12 | 1.17 |
| α-Ketoisocaproate | 1.12 | 1.15 |
| 2-Oxoglutarate | 1.19 | 1.26 |
| Threonine | 0.88 | 0.94 |
| Lactate | 0.86 | 0.84 |
| α-D-Xylp | 0.85 | 0.76 |
| α-Arabinose | 0.80 | 0.75 |
| Succinate | 0.77 | 0.85 |
| Choline | 0.76 | 0.83 |
| Pyruvate | 0.84 | 1.78 |
| Imidazole | 0.93 | 1.03 |
| Malate | 1.12 | 1.21 |
| Cytidine | 0.90 | 0.95 |
| Uridine | 0.82 | 0.91 |
| Taurine | 0.95 | 0.82 |

Fold change with a value > 1 indicates a relatively lower concentration present in the rats from CD group in each cross-comparison. Abbreviations: α-D-Xylp, α-D-xylopyranosyl.
